# BCAA mediated microbiota-liver-heart crosstalk regulates diabetic cardiomyopathy via FGF21

**DOI:** 10.1186/s40168-024-01872-3

**Published:** 2024-08-24

**Authors:** Hong Zheng, Xi Zhang, Chen Li, Die Wang, Yuying Shen, Jiahui Lu, Liangcai Zhao, Xiaokun Li, Hongchang Gao

**Affiliations:** 1 Oujiang Laboratory, School of Pharmaceutical Sciences, Wenzhou Medical University, Wenzhou, 325035 China; 2 Institute of Aging, Key Laboratory of Alzheimer’s Disease of Zhejiang Province, Wenzhou Medical University, Wenzhou, 325035 China

**Keywords:** BCAA, Diabetes, Fibrosis, Mitochondria, Gut microbiota, Heart

## Abstract

**Background:**

Diabetic cardiomyopathy (DCM) is one of leading causes of diabetes-associated mortality. The gut microbiota-derived branched-chain amino acids (BCAA) have been reported to play a central role in the onset and progression of DCM, but the potential mechanisms remain elusive.

**Results:**

We found the type 1 diabetes (T1D) mice had higher circulating BCAA levels due to a reduced BCAA degradation ability of the gut microbiota. Excess BCAA decreased hepatic FGF21 production by inhibiting PPARα signaling pathway and thereby resulted in a higher expression level of cardiac LAT1 via transcription factor Zbtb7c. High cardiac LAT1 increased the levels of BCAA in the heart and then caused mitochondrial damage and myocardial apoptosis through mTOR signaling pathway, leading to cardiac fibrosis and dysfunction in T1D mice. Additionally, transplant of faecal microbiota from healthy mice alleviated cardiac dysfunction in T1D mice, but this effect was abolished by FGF21 knockdown.

**Conclusions:**

Our study sheds light on BCAA-mediated crosstalk among the gut microbiota, liver and heart to promote DCM and FGF21 serves as a key mediator.

Video Abstract

**Supplementary Information:**

The online version contains supplementary material available at 10.1186/s40168-024-01872-3.

## Background

Cardiomyopathy occurs in both types of diabetes and is a leading cause of mortality in diabetic patients [1]. In the early stage, diabetic cardiomyopathy (DCM) manifests as cardiac hypertrophy, fibrosis and diastolic dysfunction, but systolic dysfunction and heart failure will occur in the late stage [2]. The potential mechanisms underlying the onset and development of DCM have been proposed mainly including metabolic disorder, mitochondrial injury, oxidative stress, inflammation, autophagy and fibrosis [2–4]. Of note, when each 1% increase in HbA1c level, type 1 diabetic (T1D) patients have a 30% increased risk of heart failure and type 2 diabetic (T2D) patients have a 8% increased risk [3], suggesting T1D exhibits a higher risk of heart failure than T2D. Moreover, cardiac dysfunction has presented in the early stage of T1D in children and adolescents [5]. Thus, T1D has a longer duration of DCM relative to T2D, but now most of studies regarding DCM focused on T2D. It is an urgent need to explore potential pathophysiological mechanisms of DCM caused by T1D and develop its potential therapeutic strategy.

Accumulating evidence reveals that the gut microbiota and its metabolites have been closely associated with the onset and development of cardiovascular diseases [6, 7]. Trimethylamine *N*-oxide (TMAO), an oxidation product of the gut microbial metabolite trimethylamine (TMA), was first proved to promote atherosclerosis by Wang et al. [8] Subsequently, TMAO was used as a diagnostic or prognostic metabolic biomarker for heart diseases [9–11]. Short-chain fatty acids (SCFAs), derived from the microbial fermentation, have been reported to affect hypertension and heart failure [12–14]. Bile acids, which are synthesized from hepatic cholesterol and metabolized by the gut microbiota, were also implicated in heart diseases, such as arrhythmias [15], cardiomyopathy [16] and heart failure [17]. Moreover, the gut microbiota-derived BCAA have the ability to modulate cardiac physiology and affect heart diseases [18], such as heart failure [19], ischemic heart disease [20] and cardiac hypertrophy [21]. Tobias et al. revealed that BCAA might be the central metabolic link between T2D and cardiovascular diseases. [22] In recent years, although there is growing interest in the role of the gut microbiota in the onset and progress of DCM [23], the pathophysiological mechanisms underlying the relationship between the gut microbiota, host metabolism and DCM are still far from being clearly understood.

Herein we found that T1D mice with DCM exhibited a lower BCAA degradation ability of the gut microbiota, resulting in an increased BCAA intake from the gut to the circulation. Excess circulating BCAA decreased the PPARα-mediated expression of FGF21 in the liver and thereby led to an increased expression level of cardiac BCAA transporter LAT1 via transcription factor Zbtb7c. LAT1 induced the increase of BCAA in the heart, and BCAA activated mTOR signaling and further increased cardiac LAT1 level. Excess cardiac BCAA activated mTOR signaling, caused mitochondrial damage and myocardial cell apoptosis, and contributed to cardiac fibrosis and dysfunction in T1D mice. Furthermore, we revealed that faecal microbiota transplantation from healthy mice can significantly alleviate cardiac dysfunction in T1D mice but this beneficial effect was abolished by FGF21 knockdown. Our study not only elucidates a novel mechanism on the gut microbiota-derived BCAA that drives the communication between microbiota, liver and heart and affects DCM via FGF21, but also provides a potential therapeutic strategy for DCM by targeting the gut-liver-heart axis.

## Results

Increased level of BCAA contributes to cardiac dysfunction in T1D mice.

In this study, we treated C57BL/6 male mice with multiple-low-dose STZ to develop T1D mouse model (Figure S1a). STZ-treated mice displayed typical T1D symptoms including significantly higher levels of blood glucose (Figure S1b), daily food intake (Figure S1c) and daily water intake (Figure S1d) but lower body weight (Figure S1e) compared with normal control (Ctrl) mice. After an 8-week diabetes progression, we assessed cardiac functions in mice using echocardiography (Figure S1f), and observed that T1D mice exhibited significantly lower levels of left ventricular ejection fraction (%EF, Figure S1g) and fractional shortening (%FS, Figure S1h) but higher left ventricular internal diameter at end-systole (LVIDs, Figure S1i) relative to Ctrl mice, suggesting an impaired cardiac functions in T1D mice. In addition, the size of cardiomyocytes (Figure S1j; Figure S1k) and degree of fibrosis (Figure S1j; Figure S1l) were also significantly increased in the heart of T1D mice. To develop an in vitro model, we cultured H9c2 myocardial cells under the high glucose condition (HG, 33 mM glucose) for 48 h (Figure S2a). Treatment with HG significantly increased H9c2 cell apoptosis compared with the normal glucose (NG, 5.5 mM glucose) condition (Figures S2b-S2e).

**Fig. 1:**
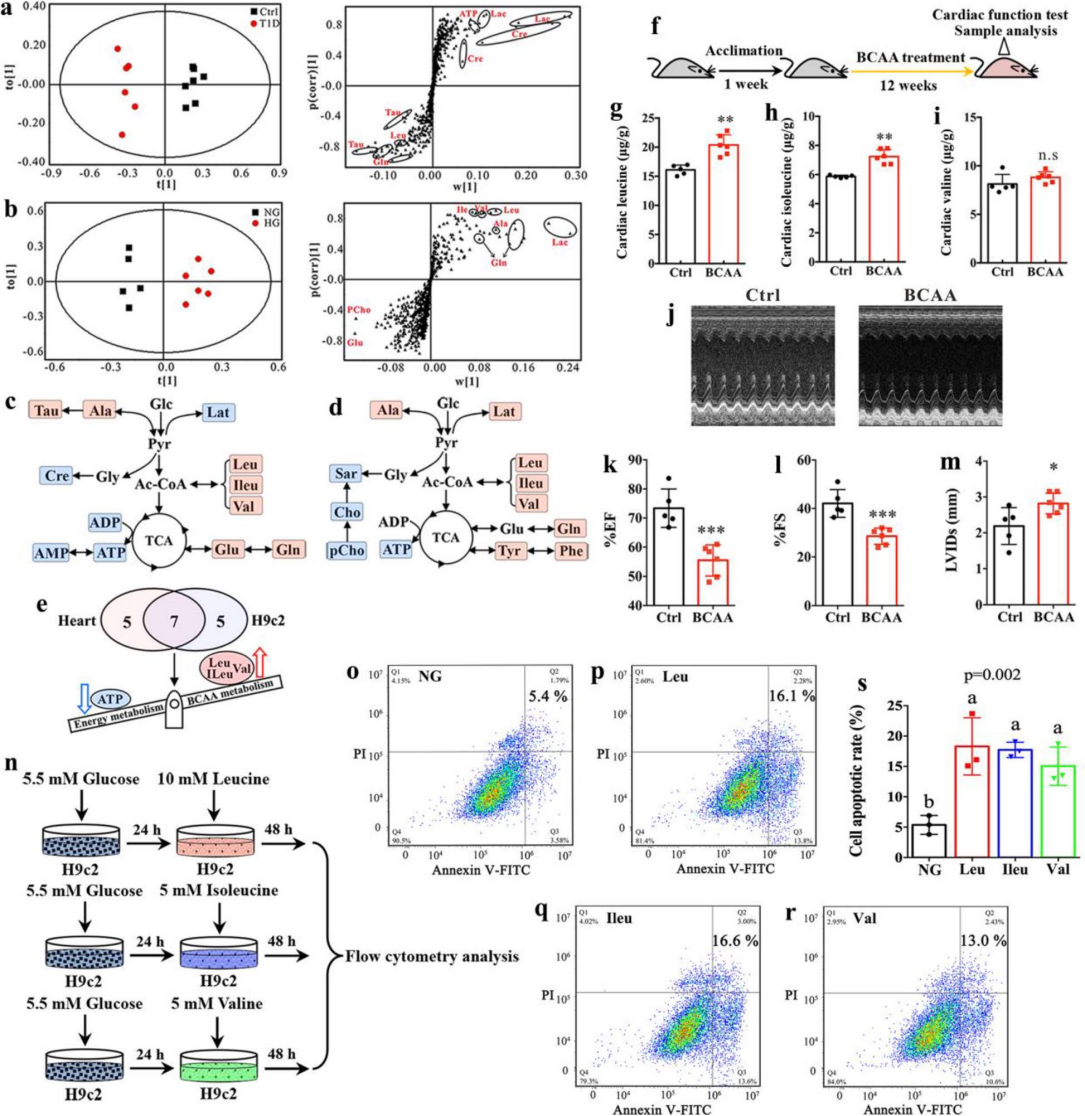
Excess BCAA causes myocardial apoptosis and cardiac dysfunction in T1D mice. OPLS-DA classification based on metabolomics data in (**a**) the heart between control (Ctrl) and type 1 diabetic (T1D) mice and **b** the myocardial cells between normal and high glucose conditions (NG and HG) and the corresponding S-plot to identify key metabolites. Altered metabolic pathways in (**c**) the heart of T1D mice and **d** HG-treated H9c2 cells; Red and blue shadings represent increase and decrease in model group relative to normal control group, respectively. **e** Common metabolic features in the heart of T1D mice and HG-treated H9c2 cells including increased BCAA metabolism and decreased energy metabolism. **f** Flow diagram of experiment: After 1 week of acclimation, mice were given BCAA in their drinking water daily for 12 weeks, and then subjected to cardiac function test with echocardiography and sample analysis (*n* = 5–6 mice per group). The concentrations of (**g**) leucine, (**h**) isoleucine and **i** valine in the heart of mice after BCAA treatment. **j** Representative M-mode echocardiographs in control (Ctrl) and BCAA-treated mice. **k** Left ventricular ejection fraction (%EF), (**l**) left ventricular fractional shortening (%FS) and **m** left ventricular internal dimension at systole (LVIDs) in Ctrl and BCAA-treated mice. **n** H9c2 cells were cultured under a high BCAA condition (10mMleucine, 5 mM isoleucine or 5 mM valine) for 48 h and then collected for sample analysis. Flow cytometry analysis in H9c2 cells under **o** normal glucose (NG), **p** 10mMleucine (Leu), **q** 5 mM isoleucine(Ileu) and **r** 5 mM valine conditions and **s** the corresponding quantitative data. Abbreviation: Leu, leucine; Ileu, isoleucine; Val, valine; Ala, alanine; Cre, creatine; Cho, choline; pCho, phosphocholine; Sar, sarcosine; Lac, lactate; Tau, taurine; Glu, glutamate; Gln, glutamine; Tyr, tyrosine; Phe, phenylalanine. The difference between two groups was analyzed by using two-tailed unpaired student’s t test with Bonferroni correction. Significant level: *p < 0.05; **p < 0.01; ***p < 0.001.The differences among three groups were analyzed by using one-way ANOVA with Bonferroni’s multiple comparisons test, and different lowercase codes represent a statistically significant difference (*p* < 0.05)

To examine the metabolic changes in both in vivo and in vitro models of the diabetic heart, an untargeted ^1^H NMR-based metabolomics analysis was carried out. Typical NMR spectra of heart tissue and H9c2 cells are illustrated in Figure S3, where a total of 30 metabolites were identified mainly involving amino acid metabolism, energy metabolism, choline metabolism, nucleoside metabolism and others. Using OPLS-DA, we found that the metabolic patterns of T1D mice or HG-treated H9c2 cells were clearly separated from those of their corresponding control groups (Fig. 1a and b). Moreover, important metabolites that mainly contributed to metabolic pattern differences were identified via S-plots. The relative concentrations of metabolites were quantified in heart tissues (Table S1) and significantly altered metabolites were presented in metabolic pathways as shown in Fig. 1c. Compared with Ctrl mice, T1D mice exhibited significantly increased levels of leucine, isoleucine, valine, alanine, glutamate, glutamine and taurine in the heart, mainly involving amino acid metabolism. However, the levels of energy metabolism-related metabolites, such as ATP, ADP, AMP, creatine and lactate, were significantly reduced in the heart of T1D mice relative to Ctrl mice. Moreover, Fig. 1b reveals that HG-induced shifts in metabolic patterns of H9c2 cells might be attributed to a series of metabolites including leucine, isoleucine, valine, alanine, glutamate, glutamine, lactate and phosphocholine. The relative concentrations of metabolites in H9c2 cells were quantified as listed in Table S2, where we found significantly higher levels of leucine, isoleucine, valine, alanine, glutamine, tyrosine, phenylalanine and lactate but lower levels of ATP, choline, phosphocholine and sarcosine in H9c2 cells under the HG condition, mainly involving in amino acid metabolism, energy metabolism and choline metabolism (Fig. 1d). Together, higher BCAA levels and lower ATP level were identified as the common metabolic characteristics in both in vivo and in vitro models of diabetes (Fig. 1e).

Then mice were given BCAA in their drinking water daily for 12 weeks to examine the impact of excess BCAA on cardiac functions in mice (Fig. 1f). We found that cardiac BCAA levels were elevated in mice after BCAA administration (Fig. 1g-1i) especially leucine (Fig. 1g) and isoleucine (Fig. 1h). Mice receiving oral BCAA supplementation showed an impaired cardiac functions (Fig. 1j), as indicated by decreases in %EF (Fig. 1k) and %FS (Fig. 1l) and an increase in LVIDs (Fig. 1m). To investigate whether increased BCAA levels cause myocardial apoptosis, we cultured H9c2 cells with leucine (10 mM), isoleucine (5 mM) or valine (5 mM) for 48 h (Fig. 1n). The results from flow cytometry demonstrate that higher levels of all three BCAA significantly increased H9c2 cell apoptosis relative to the low-BCAA condition (Fig. 1o-s), indicating the adverse effects of excess BCAA on myocardial cells. Taken together, our results suggest that increased level of BCAA contributed to myocardial damage and cardiac dysfunction in T1D mice.

### Elevated BCAA derives from reduced BCAA degradation ability in the gut microbiota of T1D mice

**Fig. 2:**
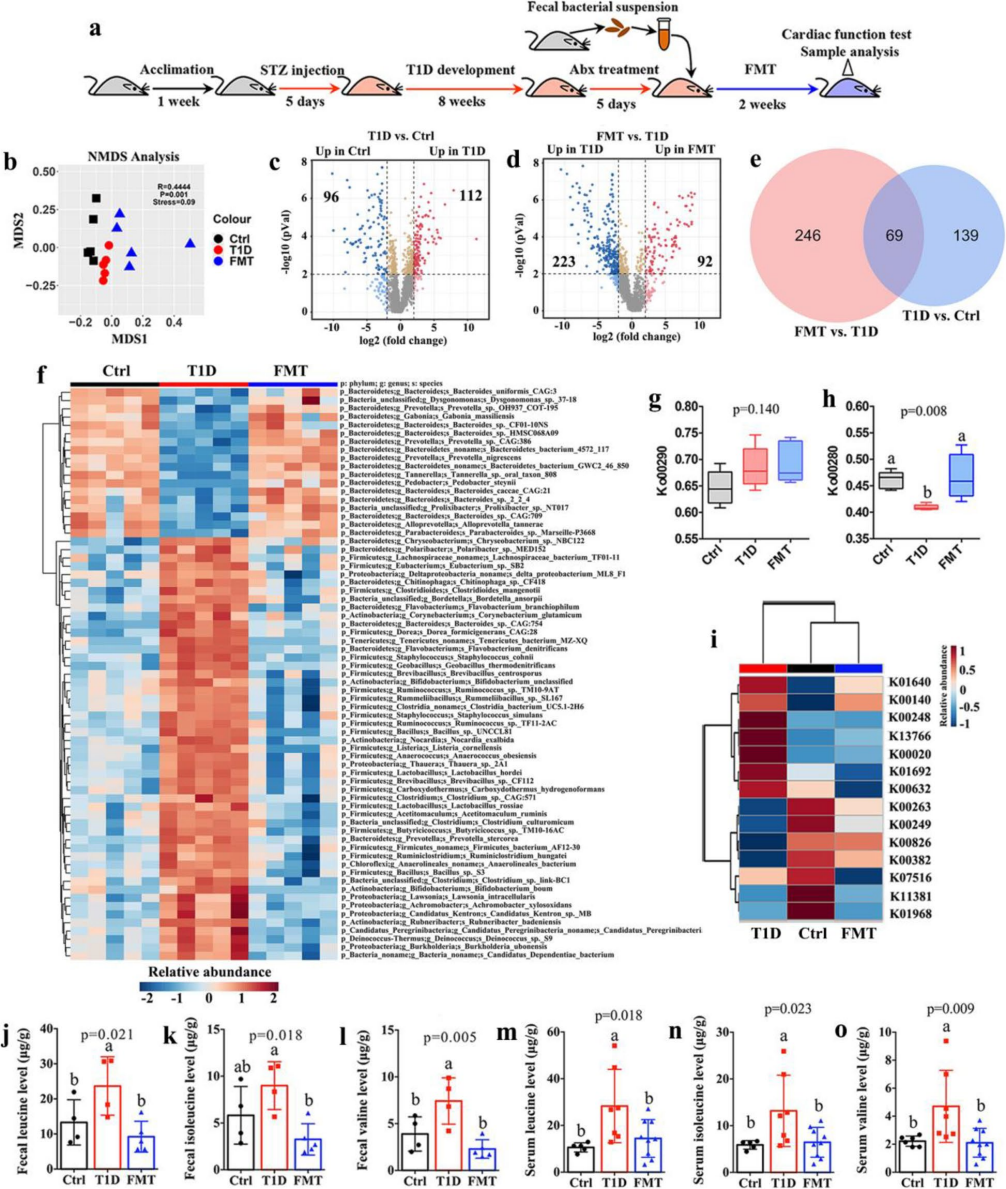
Elevated BCAA derive from reduced BCAA degradation ability of the gut microbiota in T1D mice. **a** Flow diagram of FMT experiment: After 1 week of acclimation, mice were injected with streptozocin (STZ) for 5 days to develop type 1 diabetic (T1D) mice and then administered with a 5-day antibiotic (Abx) treatment after 8 weeks. Subsequently, faecal material from control (Ctrl) mice was transferred to Abx-treated T1Dmice (FMT) for 2 weeks. Finally, mice were subjected to cardiac function test with echocardiography and sample analysis (*n* = 5 mice per group). **b** NMDS analysis showing the beta diversity of the gut microbiota in Ctrl, T1D and FMT mice. Volcano plot analysis based on the gut microbiota at the species level (**c**) between Ctrl and T1D mice and **d** between T1D and FMT mice. **e** Venn diagram showing 69 gut microbes that were significantly altered in T1D mice relative to Ctrl mice and then also varied after FMT. **f** Heatmap showing changes in 69 gut microbes identified from volcano plot. **g** BCAAs biosynthesis (Ko00290) and **h** BCAAs degradation (Ko00280) of the gut microbiota in Ctrl, T1D and FMT mice. **i** The levels of key enzymes in BCAA degradation of the gut microbiota among Ctrl, T1D and FMT mice. The levels of (**j**, **m**) leucine, (**k**, **n**) isoleucine and **l**, **o** valine in the feces and serum of Ctrl, T1D and FMT mice. The differences among three groups were analyzed by using one-way ANOVA with Bonferroni’s multiple comparisons test, and different lowercase codes represent a statistically significant difference (*p* < 0.05)

Since BCAA are derived from the gut microbiota and exert potential influences on metabolic disease [24, 25], the changes in the gut microbiome were analyzed by using metagenomics analysis and transplanted the gut microbiota from healthy mice to T1D mice in order to explore the impact of the gut microbiota on host BCAAs levels (Fig. 2a). We found that the taxon richness (observed species) of the gut microbiota was decreased in T1D mice after FMT (Figure S4a, *p* = 0.005), but no significant alteration in the taxon diversity, Shannon index (Figure S4b, *p* = 0.168). At the phylum level, T1D mice had higher percentages of *Firmicutes*, *Proteobacteria* and *Actinobacteria* but lower percentage of *Bacteroidetes* than Ctrl mice, while these changes can be reversed in T1D mice after FMT (Figures S4c-S4e). NMDS analysis shows that the beta diversity of the gut microbiota altered significantly in T1D mice after FMT (Fig. 2b). Furthermore, we identified 69 gut microbes at the species level that significantly altered in T1D mice relative to Ctrl mice but reversed after FMT via volcano plot and Venn diagram analyses (Fig. 2c-e). Figure 2f shows that T1D-induced shifts in the gut microbiota were significantly recovered after FMT, including a series of gut microbes involved in BCAA metabolism such as *Prevotella_sp._OH937_COT-195, Prevotella_sp._CAG:386, Prevotella_nigrescens* and *Prevotella_stercorea* at the *Prevotella* genus, *Bacteroides_uniformis_CAG:3, Bacteroides_sp._CF01-10NS, Bacteroides_sp._HMSC068A09, Bacteroides_caccae_CAG:21, Bacteroides_sp._2_2_4, Bacteroides_sp._CAG:709* and *Bacteroides_sp._CAG:754* at the *Bacteroides* genus, *Eubacterium_sp._SB2* at the *Eubacterium* genus as well as *Dorea_formicigenerans_CAG:28* at the *Dorea*genus [26].

To further examine the changes of the microbial functions, we annotated the metagenomic reads according to the KEGG database. Figure S5 illustrates that T1D mice after FMT were clustered together with Ctrl mice but clearly differed from T1D mice, suggesting that FMT reversed T1D-induced shifts in the microbial functions. The results reveal that there was no significant difference in BCAA biosynthesis (ko00290, Fig. 2g), while BCAA degradation was markedly reduced in T1D mice relative to Ctrl mice but significantly increased after FMT (ko00280, Fig. 2h). Moreover, we found that in the levels of four key enzymes in BCAA degradation were recovered in T1D mice after FMT such as leucine dehydrogenase (K00263), acyl-CoA dehydrogenase (K00249), BCAA aminotransferase (K00826) and dihydrolipoamide dehydrogenase (K00382) (Fig. 2i). The levels of leucine, isoleucine and valine were also detected to be significantly decreased in T1D mice after FMT in feces (Fig. 2j-l) and serum (Fig. 2m-o). Therefore, increased levels of BCAA in T1D mice might be attributed to reduced BCAA degradation ability of the gut microbiota.

### High LAT1 expression leads to increased levels of BCAA in myocardial cells

To inspect whether increased BCAA levels derive from reduced metabolic clearance, we analyzed the gene expression levels of their key metabolic enzymes. The results show that higher expression levels of *BCAT, BCKD, BCKDK* and *PP2Cm*were detected in both HG-treated myocardial cells (Figure S6a) and diabetic heart (Figure S6b) compared with the corresponding control groups, indicating an increased BCAA catabolism. After HG treatment, the BCAA levels were significantly increased in H9c2 cells but decreased in the cell culture media (Figure S7a, Table S3), suggesting an enhanced BCAA transport in cardiomyocytes under the HG condition. L-type amino acid transporter 1 (LAT1) is essential for transporting neutral amino acids including BCAA into cells [27]. We found that HG-treated H9c2 cells exhibited an increased LAT1 in both mRNA (Figure S7b) and protein (Figures S7c-S7f) levels. A significantly higher LAT1 level was also detected in the heart of T1D mice than Ctrl mice (Figures S7g-S7k). Thus our results suggest that higher levels of BCAA in the diabetic heart might be due to increased LAT1 expression.

### High LAT1 expression induces myocardial apoptosis by regulating mTOR/Bax/Bcl-2/caspase-3 signaling pathway

**Fig. 3:**
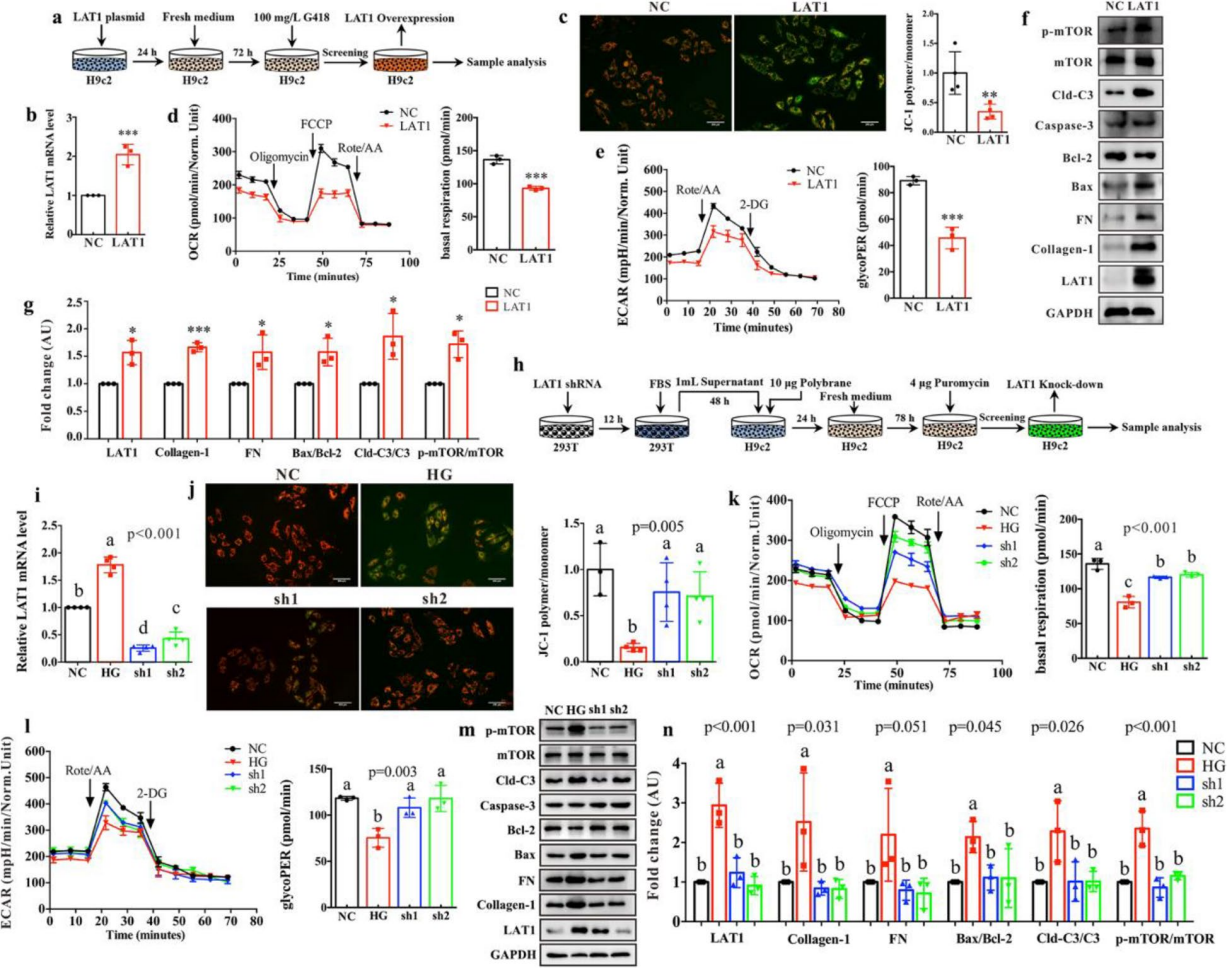
LAT1 affects mitochondrial functions and apoptosis of myocardial cells. **a** Flow diagram of LAT1 overexpression in H9c2 cells using a liposome transfection and selected stable clones after G418 screening. **b** Relative LAT1 mRNA level in normal control (NC) and LAT1-overexpressed H9c2 cells. **c** Representative images ofJC-1 staining and the ratio of JC-1 polymer/monomer to indicate the mitochondrial membrane potential in NC and LAT1-overexpressed H9c2 cells. **d** The curve of oxygen consumption rate (OCR) and the corresponding quantitative data to indicate mitochondrial respiration in NC and LAT1-overexpressed H9c2 cells. **e** The curve of extracellular acidification rate (ECAR) and the corresponding quantitative data to indicate mitochondrial glycolysis in NC and LAT1-overexpressed H9c2 cells. **f** Western blotting showing the expression levels of LAT1, Collagen-1, fibronectin (FN), Bax, Bcl-2, caspase-3, cleaved caspase-3 (Cld-C3), mTOR and p-mTOR in NC and LAT1-overexpressed H9c2 cells and **g** the corresponding quantitative data. **h** Flow diagram of LAT1 knockdown using a shRNA lentivirusin H9c2 cells and selected stable clones by puromycin. **i** Relative LAT1 mRNA level in NC, high glucose (HG) and LAT1-knockdown (sh1 and sh2) H9c2 cells. **j** Representative images ofJC-1 staining and the ratio of JC-1 polymer/monomer in NC, HG and LAT1-knockdown (sh1 and sh2) H9c2 cells. **k** The curve of OCR and the corresponding quantitative data in NC, HG and LAT1-knockdown (sh1 and sh2) H9c2 cells. **l** The curve of ECAR and the corresponding quantitative data in NC, HG and LAT1-knockdown (sh1 and sh2) H9c2 cells. **m** Western blotting showing the expression levels of LAT1, Collagen-1, fibronectin (FN), Bax, Bcl-2, caspase-3, cleaved caspase-3 (Cld-C3), mTOR and p-mTOR in NC, HG and LAT1-knockdown (sh1 and sh2) H9c2 cells and **n** the corresponding quantitative data. The difference between two groups was analyzed by using two-tailed unpaired student’s t test with Bonferroni correction. Significant level: *p < 0.05; **p < 0.01; ***p < 0.001. The differences among four groups were analyzed by using one-way ANOVA with Bonferroni’s multiple comparisons test, and different lowercase codes represent a statistically significant difference (*p* < 0.05)

It is well known that BCAA fuel the tricarboxylic acid (TCA) cycle intermediates and produce ATP for energy supply [28]. In this study, a reduced ATP level may reflect mitochondrial dysfunction in diabetic heart and HG-treated cardiomyocytes. To examine the role of LAT1 in mitochondrial functions, a liposome transfection technique was used to achieve overexpression of LAT1 gene in H9c2 cells and obtain stable clones after G418 screening (Fig. 3a). As can be seen from Fig. 3b, LAT1 gene was significantly over-expressed in H9c2 cells. The mitochondrial membrane potential (MMP) was assessed by JC-1 staining, and the ratio of JC-1 polymer/monomer was significantly decreased in LAT1-overexpressed H9c2 cells relative to normal control (NC) cells (Fig. 3c), implying that the MMP was reduced in cardiomyocytes after LAT1 overexpression. Using a seahorse analyzer, we measured oxygen consumption rate (OCR) and extracellular acidification rate (ECAR) to indicate mitochondrial respiration and glycolysis, respectively [29]. The results reveal that the levels of these two parameters were significantly decreased in LAT1-overexpressed H9c2 cells (Fig. 3d and e), which further confirmed mitochondrial dysfunction after LAT1 overexpression. Moreover, we found that the levels of collagen-1 and fibronectin (FN) as fibrotic biomarkers were significantly increased in H9c2 cells overexpressing LAT1 (Fig. 3f and g). The ratios of Bax/Bcl-2, cleaved caspase-3/caspase-3 (Cld-C3/C3) and p-mTOR/mTOR were also markedly up-regulated as shown in Fig. 3f and g.

To further determine the role of LAT1 in mitochondrial functions, we carried out LAT1 knockdown via a shRNA lentivirus and selected stable clones by puromycin, as illustrated in Fig. 3h. The relative level of LAT1 mRNA was significantly lower in H9c2 cells with LAT1 knockdown (Fig. 3i). We observed that the MMP was dramatically decreased in H9c2 cells under the HG condition relative to the normal controls, but LAT1 knockdown can effectively protect against HG-induced damage in the MMP of H9c2 cells (Fig. 3j). Additionally, HG-induced reductions in mitochondrial respiration (Fig. 3k) and glycolysis (Fig. 3l) were recovered in H9c2 cells after LAT1 knockdown. Also, myocardial fibrosis was obviously reduced in LAT1-knockdown H9c2 cells under the HG condition, as indicated by lower collagen-1 and FN levels (Fig. 3m and n). Meanwhile, LAT1 knockdown significantly diminish the ratios of Bax/Bcl-2, Cld-C3/C3 and p-mTOR/mTOR in HG-treated H9c2 cells (Fig. 3m and n).

To verify the pivotal role of mTOR in mitochondrial functions and myocardial fibrosis, rapamycin (Rapa) was used to specifically inhibit mTOR signaling in H9c2 cells (Figure S8a). We found that mTOR inhibition can alleviate the MMP damage of H9c2 cells under the HG condition (Figures S8b and S8c). HG-induced mitochondrial disorders were also relieved in H9c2 cells after inhibition of mTOR, as indicated by higher mitochondrial respiration (Figures S8d and S8e) and glycolysis (Figures S8f and S8g). The levels of two fibrosis biomarkers, collagen-1 and FN, were increased in H9c2 cells under the HG condition, but significantly reduced after mTOR inhibition (Figures S8h and S8i). The ratios of Bax/Bcl-2, Cld-C3/C3 and p-mTOR/mTOR were also effectively inhibited in Rapa-treated H9c2 cells (Figures S8h and S8i). Additionally, the inhibition of mTOR significantly reduced the LAT1 expression level (Figures S8h and S8i). Therefore, our results indicate that lower LAT1 can improve mitochondrial dysfunction and relieve apoptosis and myocardial fibrosis via the mTOR/Bax/Bcl-2/caspase-3 signaling pathway, and there was a positive feedback loop between LAT1 and mTOR (Figure S8j).

### Fecal microbiota transplant improves cardiac functions of T1D mice by reducing LAT1-driven increase in BCAA

**Fig. 4:**
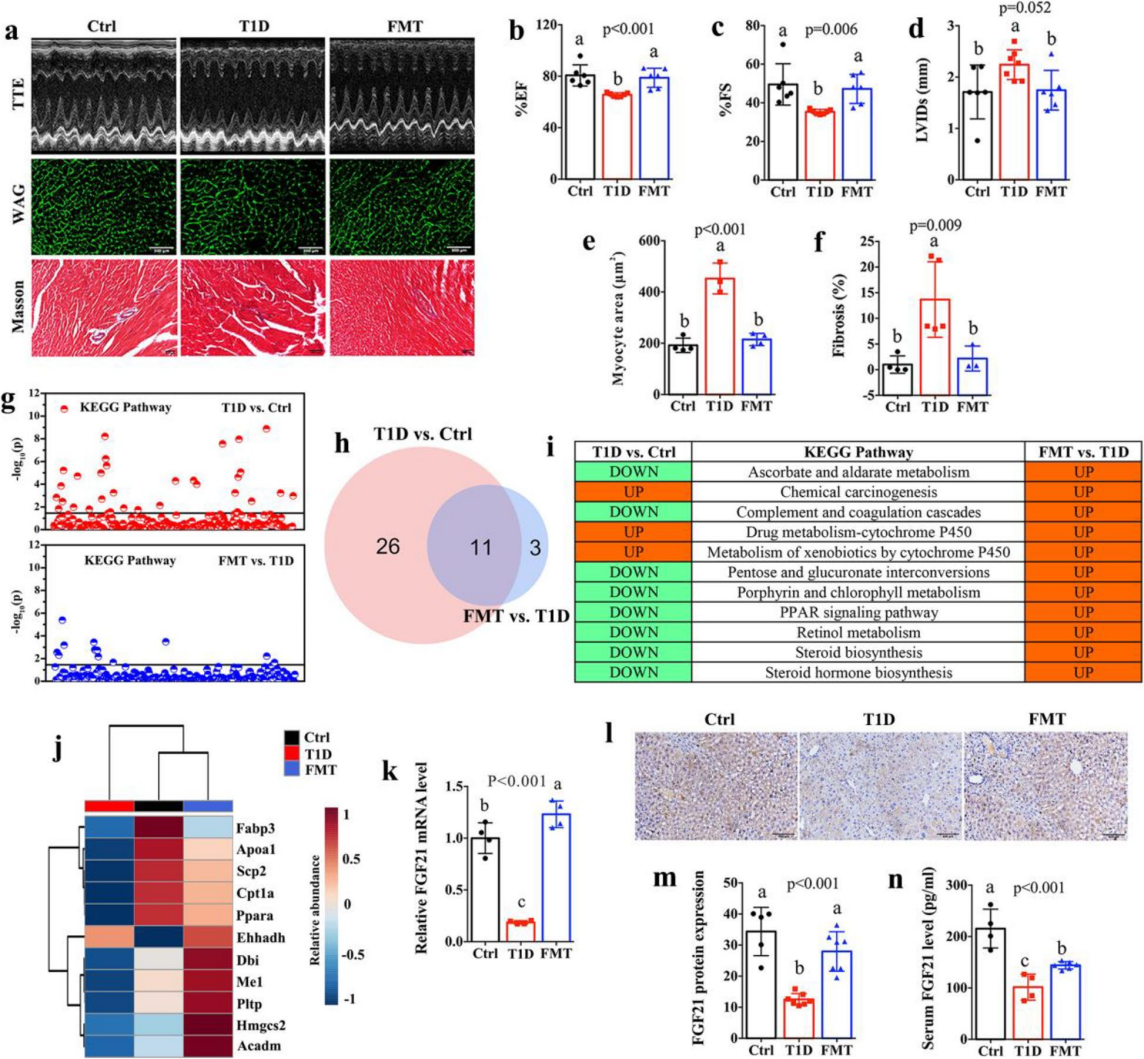
Fecal microbiota transplant improves cardiac functions and increases hepatic FGF21 production in T1D mice. **a** Representative images of M-mode echocardiographs, wheat germ agglutinin (WGA) and Masson staining (bar = 100 μm) in control (Ctrl) mice, type 1 diabetic (T1D) mice and T1D mice receiving faecal material from Ctrl mice (FMT). **b** Left ventricular ejection fraction (%EF), **c** left ventricular fractional shortening (%FS), **d** left ventricular internal dimension at systole (LVIDs), **e** cardiomyocyte size and **f** degree of fibrosis in Ctrl, T1D and FMT mice. **g** KEGG pathways altered in the liver between T1D and Ctrl mice and between FMT and T1D mice based on transcriptomic data. **h** Venn diagram showing 11 KEGG pathways that were significantly altered in T1D mice relative to Ctrl mice and then also varied after FMT. **i** Table showing the detailed information of 11 KEGG pathways. **j** Heatmap showing changes in key gene levels in PPAR signaling pathway. **k** Relative FGF21 mRNA level in the liver of Ctrl, T1D and FMT mice. **l** Representative histological images of FGF21 staining (bar = 100 μm) and **m** the corresponding quantitative data. **n** The level of FGF21 in the serum of Ctrl, T1D and FMT mice. The differences among three groups were analyzed by using one-way ANOVA with Bonferroni’s multiple comparisons test, and different lowercase codes represent a statistically significant difference (*p* < 0.05)

We assessed the effect of FMT on cardiac functions in T1D mice (Fig. 4a) and found that FMT effectively improved cardiac structure and functions in T1D mice, as indicated by significantly higher levels of EF% (Fig. 4b) and FS% (Fig. 4c) and lower level of LVIDs (Fig. 4d), the cardiomyocyte hypertrophy (Fig. 4a and e) and degree of fibrosis (Fig. 4a and f) in T1D mice after FMT. Moreover, FMT can reduce a high LAT1 level in the heart of T1D mice (Figures S9a and S9b), resulting in lower cardiac BCAA levels (Figures S9c). Atrial natriuretic peptide (ANP) and B-type natriuretic peptide (BNP) are primarily secreted by the heart and increased in patients with the cardiac dysfunction especially for heart failure [30]. Herein we observed that the levels of these two markers were significantly increased in the heart of T1D mice but decreased after FMT (Figures S9d and S9e). Two markers of myocardial fibrosis, collagen-1 and FN, were also notably lower in the heart of T1D mice after FMT (Figures S9d and S9e). Moreover, FMT can significantly inhibit the mTOR/Bax/Bcl-2/caspase-3 signaling pathway in the heart of T1D mice (Figures S9d and S9e). Thus, our results suggest that FMT improves cardiac functions in T1D mice by reducing LAT1-driven increase in BCAA and down-regulating the mTOR/Bax/Bcl-2/caspase-3 pathway.

### Excess BCAA decreases hepatic FGF21 production in T1D mice

Since the liver is the center of metabolism and the hormonal system, we analyzed the transcriptome changes in the liver of T1D mice after FMT. We identified 11 common KEGG pathways that significantly altered between T1D and Ctrl mice as well as between FMT and T1D mice (Fig. 4g and h), where PPAR signaling pathway was identified to be down-regulated in T1D mice relative to Ctrl mice but up-regulated after FMT (Fig. 4i). Furthermore, the levels of *PPARα, Cpt1a, Scp2* and *Apoa1* were significantly reduced in the liver of T1D mice compared with Ctrl mice but increased in T1D mice after FMT (Fig. 4j). PPARα signaling pathway has been reported as a key regulator of hepatic FGF21 production [31, 32]. The adverse effects of BCAA on metabolic health might be mediated by FGF21 [33, 34]. Thus, we measured hepatic expression of FGF21 and circulating FGF21 level in T1D mice after FMT. The results demonstrate that T1D mice has a significantly lower FGF21 level in the liver and serum than Ctrl mice and this effect can be reversed in T1D mice with FMT (Fig. 4k-n). Interestingly, the levels of *FGF21* and *PPARα* were drastically reduced in the liver of mice receiving oral BCAA supplementation (Figure S10a) and the hepatocyte cell line AML-12 under high BCAA condition (Figure S10b). These findings indicate that excess BCAA can decrease the PPARα-mediated expression of FGF21 in the liver.

### FGF21 suppresses LAT1 expression and protects against cardiac dysfunction in T1D mice

**Fig. 5:**
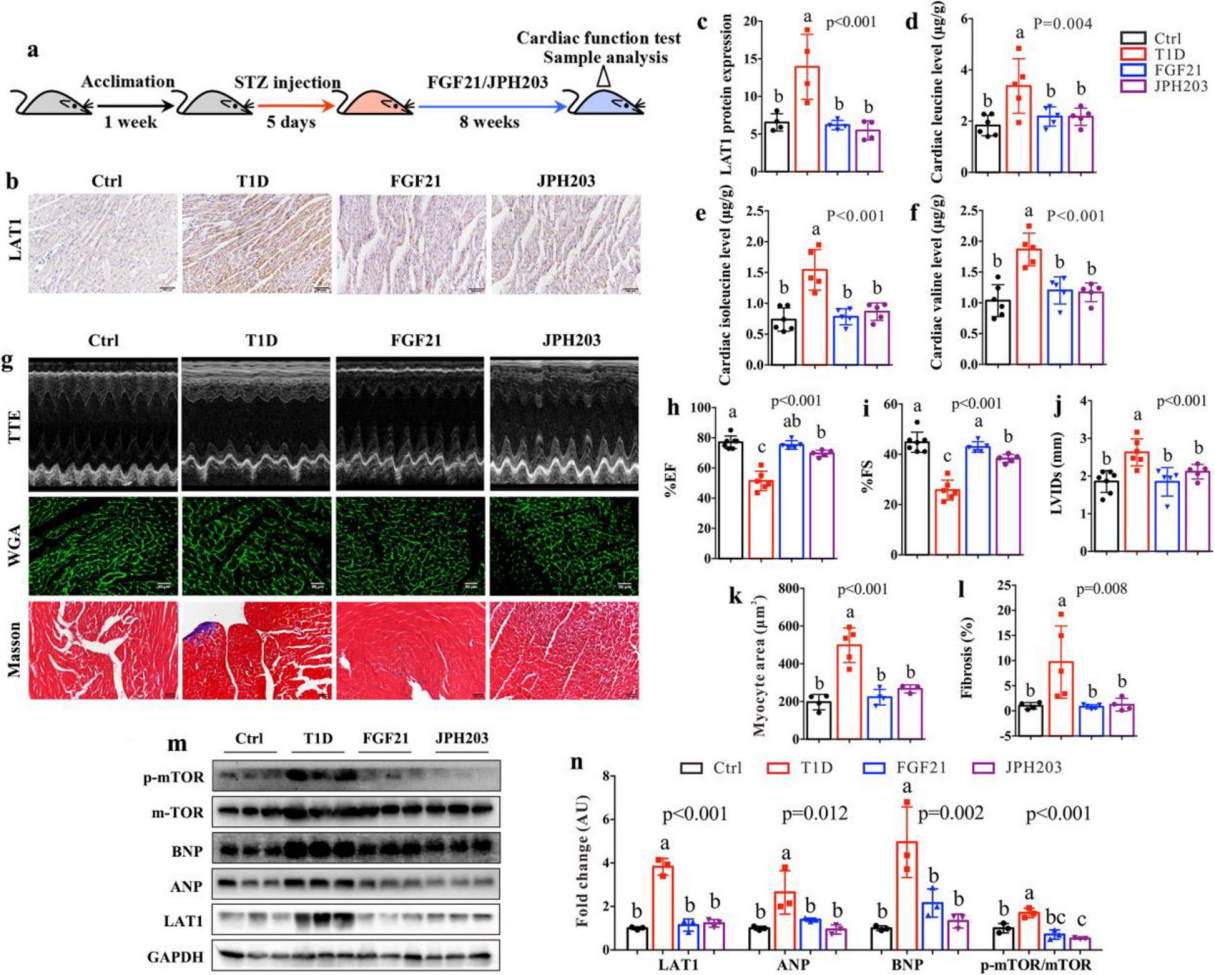
FGF21 inhibits LAT1 and improves cardiac dysfunction in T1D mice. **a** Flow diagram of experiment: After 1 week of acclimation, mice were injected with streptozocin (STZ) for 5 days to induce type 1 diabetic (T1D) mice and then treated with FGF21 or JPH203 as a positive control for 8 weeks. After 8 weeks, mice were subjected to cardiac function test with echocardiography and sample analysis (*n* = 5–7 mice per group). **b** Representative histological images of LAT1 staining (bar = 100 μm) and **c** the corresponding quantitative data to show the level of LAT1 in the heart of normal control (Ctrl), T1D, FGF21-treated and JPH203-treated mice. The levels of **d** leucine, **e** isoleucine and **f** valine in the heart of Ctrl, T1D, FGF21-treated and JPH203-treated mice. **g** Representative images of M-mode echocardiographs, wheat germ agglutinin (WGA) and Masson staining (bar = 100 μm) in Ctrl, T1D, FGF21-treated and JPH203-treated mice. **h** Left ventricular ejection fraction (%EF), **i** left ventricular fractional shortening (%FS), **j** left ventricular internal dimension at systole (LVIDs), **k** cardiomyocyte size and **l** degree of fibrosis in Ctrl, T1D, FGF21-treated and JPH203-treated mice. **m** Western blotting showing the expression levels of LAT1, atrial natriuretic peptide (ANP), B-type natriuretic peptide (BNP), mTOR and p-mTOR in Ctrl, T1D,FGF21-treated and JPH203-treated mice and **n** the corresponding quantitative data. The differences among four groups were analyzed by using one-way ANOVA with Bonferroni’s multiple comparisons test, and different lowercase codes represent a statistically significant difference (*p* < 0.05)

To explore the effect of FGF21 on cardiac dysfunction induced by higher levels of LAT1 and BCAA, we treated T1D mice with FGF21 for 8 weeks (Fig. 5a). Besides, JPH203, as a selective inhibitor of LAT1 [35], was used as a positive control. The results reveal that FGF21, like JPH203, significantly inhibited cardiac LAT1 protein expression (Fig. 5b and c), resulting in lower levels of leucine (Fig. 5d), isoleucine (Fig. 5e) and valine (Fig. 5f) in the heart of T1D mice. Treatment with FGF21 or JPH203 markedly increased levels of EF% (Fig. 5g and h) and FS% (Fig. 5g and i) and decreased levels of LVIDs (Fig. 5g and j),cardiomyocyte hypertrophy (Fig. 5g and k) and fibrosis (Fig. 5g and l) in T1D mice, suggesting that FGF21 can improve diabetes-associated cardiac structure damage and dysfunction. We also found that the levels of ANP and BNP were dramatically reduced in the heart of T1D mice after FGF21 or JPH203 treatment (Fig. 5m and n). In addition, both FGF21 and JPH203 significantly inhibited the mTOR signaling pathway in the heart of T1D mice (Fig. 5m and n). These findings indicate that the positive feedback loop between LAT1 and mTOR can be regulated by FGF21 and JPH203, but the detailed mechanisms still need to be further investigated.

To examine the impact of FGF21 on mitochondrial functions and myocardial fibrosis, we treated H9c2 cells with FGF21 or JPH203, as illustrated in Figure S11a. The results reveal that treatment with FGF21 or JPH203 significantly reduced the MMP damage of H9c2 cells under the HG condition (Figures S11b and S11c). Mitochondrial dysfunction induced by HG was also relieved in H9c2 cells after FGF21 or JPH203 treatment, as indicated by increased mitochondrial respiration (Figures S11d and S11e) and glycolysis (Figures S11f and S11g). Additionally, HG-induced increases in collagen-1 and FN were notably suppressed in H9c2 cells with FGF21 or JPH203 treatment (Figures S11h and S11i). Our results suggest that the beneficial effect of FGF21 might be achieved by reducing LAT1-driven increase in BCAA (Figure S12) and thereby inhibiting the mTOR/Bax/Bcl-2/caspase-3 signaling pathway (Figures S11h and S11i).

**Fig. 6:**
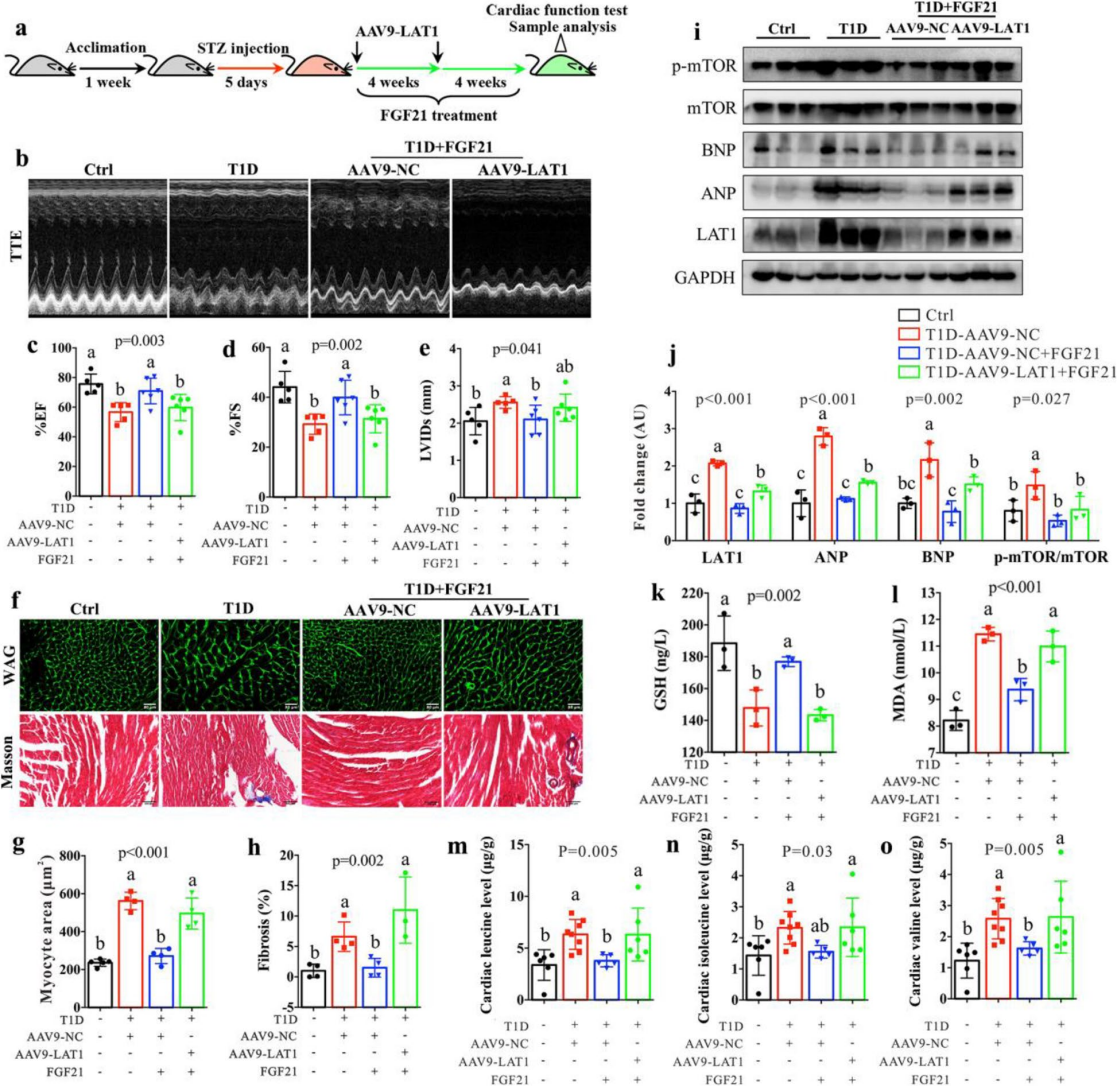
AAV-mediated LAT1 overexpression abolishes the cardioprotective effect of FGF21 in T1D mice. **a** Flow diagram of experiment: After 1 week of acclimation, mice were injected with streptozocin (STZ) for 5 days to induce type 1 diabetic (T1D) mice and then treated with AAV9-LAT1 for the specific overexpression of LAT1 in the heart of T1D mice during FGF21 treatment for 8 weeks. After 8 weeks, mice were subjected to cardiac function test with echocardiography and sample analysis (*n* = 5–6 mice per group). **b** Representative M-mode echocardiographs in control (Ctrl) and T1D mice as well as T1D mice treated with FGF21 plus AAV9-NC (empty vector) or AAV9-LAT1. **c** Left ventricular ejection fraction (%EF), **d** left ventricular fractional shortening (%FS) and **e** left ventricular internal dimension at systole (LVIDs) in Ctrl and T1D mice and T1D mice treated with FGF21 plus AAV9-NC or AAV9-LAT1. **f** Representative histological images of wheat germ agglutinin (WGA) and Masson staining (bar = 100 μm) and the corresponding quantitative data to show the changes of **g** cardiomyocyte size and **h** degree of fibrosis in Ctrl and T1D mice and T1D mice treated with FGF21 plus AAV9-NC or AAV9-LAT1. **i** Western blotting showing the expression levels of LAT1, atrial natriuretic peptide (ANP), B-type natriuretic peptide (BNP), mTOR and p-mTOR in Ctrl and T1D mice and T1D mice treated with FGF21 plus AAV9-NC or AAV9-LAT1 and **j** the corresponding quantitative data. The levels of **k** reduced glutathione (GSH) and **l** malondialdehyde (MDA) in the heart of Ctrl and T1D mice and T1D mice treated with FGF21 plus AAV9-NC or AAV9-LAT1. The levels of **m** leucine, **n** isoleucine and **o** valine in the heart of Ctrl and T1D mice and T1D mice treated with FGF21 plus AAV9-NC or AAV9-LAT1. The differences among four groups were analyzed by using one-way ANOVA with Bonferroni’s multiple comparisons test, and different lowercase codes represent a statistically significant difference (*p* < 0.05)

To further confirm the essential role of LAT1 in FGF21-mediated improvement in cardiac functions, AAV9-LAT1 was used for the specific overexpression of LAT1 in the heart of T1D mice during FGF21 treatment (Fig. 6a). We found that FGF21 treatment significantly increased the levels of EF% (Fig. 6b and c) and FS% (Fig. 6b and d) and reduced the level of LVIDs (Fig. 6b and e) in T1D mice, suggesting improved cardiac functions. However, of note, these positive effects of FGF21 were suppressed after LAT1 overexpression. FGF21-mediated improvements in cardiomyocyte hypertrophy (Fig. 6f and g) and fibrosis (Fig. 6f and h) were also restrained in T1D mice after LAT1 overexpression. The levels of ANP and BNP were significantly reduced in the heart of T1D mice after FGF21 treatment, but increased following LAT1 overexpression (Fig. 6i and j). Besides, we measured reduced glutathione (GSH) and malondialdehyde (MDA) to evaluate mitochondrial oxidative status in the heart [36]. The results show that treatment with FGF21 can protect cardiac mitochondria in T1D mice from oxidative damage as indicated by a significantly increased GSH (Fig. 6k) and decreased MDA (Fig. 6l), but these beneficial effects of FGF21 were significantly restrained in the heart of T1D mice after LAT1 overexpression. Additionally, we found that the overexpression of LAT1 notably boosted LAT1-driven increase in BCAA (Fig. 6m-o) and activated mTOR signaling pathway (Fig. 6i and j).

### Hepatic FGF21 knockdown abolishes the protective effect of fecal microbiota transplant on cardiac functions in T1D mice

**Fig. 7:**
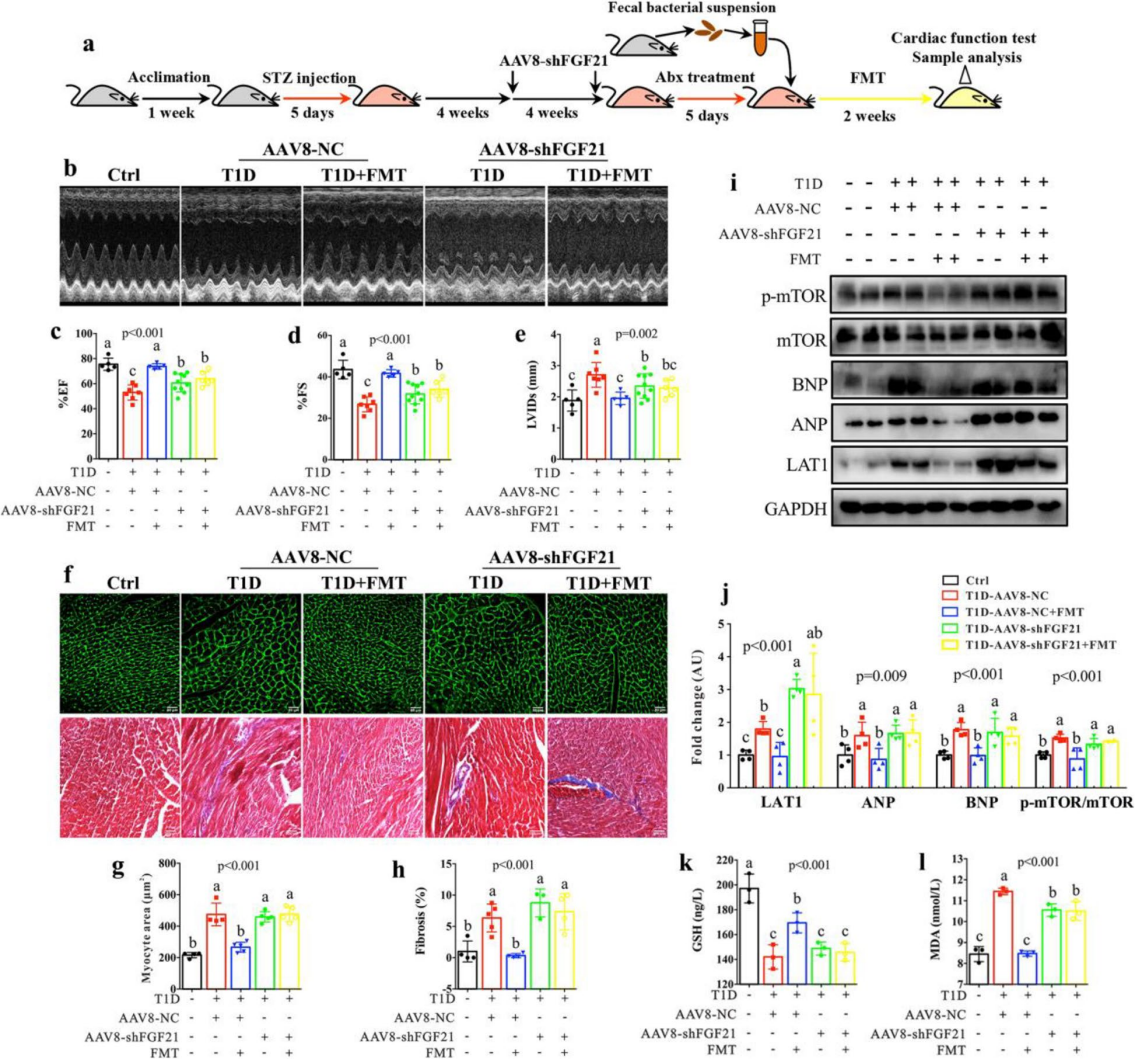
AAV-mediated FGF21 knockdown suppresses the cardioprotective effect of FMT in T1D mice. **a** Flow diagram of experiment: After 1 week of acclimation, mice were injected with streptozocin (STZ) for 5 days to induce type 1 diabetic (T1D) mice, treated with AAV8-shFGF21 for the liver-specific knockdown of FGF21 and then carried out FMT for 2 weeks. Subsequently, mice were subjected to cardiac function test with echocardiography and sample analysis (*n* = 5–10 mice per group). **b** Representative M-mode echocardiographs in control (Ctrl) and T1D mice treated with AAV8-NC (empty vector) or AAV8-shFGF21 with and without FMT. **c** Left ventricular ejection fraction (%EF), **d** left ventricular fractional shortening (%FS) and **e** left ventricular internal dimension at systole (LVIDs) in Ctrl and T1D mice treated with AAV8-NC or AAV8-shFGF21 with and without FMT. **f** Representative histological images of wheat germ agglutinin (WGA) and Masson staining (bar = 100 μm) and the corresponding quantitative data to show the changes of **g** cardiomyocyte size and **h** degree of fibrosis in Ctrl and T1D mice treated with AAV8-NC or AAV8-shFGF21 with and without FMT. **i** Western blotting showing the expression levels of LAT1, atrial natriuretic peptide (ANP), B-type natriuretic peptide (BNP), mTOR and p-mTOR in Ctrl and T1D mice treated with AAV8-NC or AAV8-shFGF21 with and without FMT and **j** the corresponding quantitative data. The levels of **k** reduced glutathione (GSH) and **l** malondialdehyde (MDA) in the heart of Ctrl and T1D mice treated with AAV8-NC or AAV8-shFGF21 with and without FMT. The differences among four groups were analyzed by using one-way ANOVA with Bonferroni’s multiple comparisons test, and different lowercase codes represent a statistically significant difference (*p* < 0.05)

Subsequently, we considered two aspects of reasons: firstly, the liver is the main source organ of FGF21 [37]; secondly, AAV8 is a specific vector for liver-directed gene editing [38]. Therefore, to investigate the essential role of FGF21 in the regulation of cardiac functions by the gut microbiota, we used AAV8-shFGF21 to achieve the liver-specific knockdown of FGF21 in T1D mice and then carried out FMT for 2 weeks, as illustrated in Fig. 7a. The results show that the level of FGF21 protein was dramatically reduced in the liver of T1D mice after AAV8-shFGF21 treatment, suggesting that the liver-specific knockdown of FGF21 was effective (Figures S13a-S13d). In addition, we found that FMT significantly increased hepatic FGF21 level in T1D mice treated with AAV8-NC (empty vector), but not in AAV8-shFGF21-treated T1D mice (Figures S13a-S13d). After FMT, cardiac functions can be improved in AAV8-NC-treated T1D mice, as indicated by significantly higher EF% (Fig. 7b and c) and FS% (Fig. 7b and d) and lower LVIDs (Fig. 7b and e), but not in AAV8-shFGF21-treated T1D mice. FMT significantly decreased the cardiomyocyte hypertrophy (Fig. 7f and g) and fibrosis (Fig. 7f and h) in T1D mice and this effect was suppressed after FGF21 knockdown. The levels of cardiac ANP and BNP were markedly decreased in T1D mice after FMT, but no significant effect was obtained due to the knockdown of FGF21 (Fig. 7i and j). Moreover, we found that mitochondrial oxidative damage can be effectively alleviated in the heart of T1D mice following FMT, as indicated by significantly increased GSH level (Fig. 7k) and decreased MDA level (Fig. 7l), while this phenomenon was not observed in FGF21-knockdown T1D mice. FMT reduced the levels of LAT1 (Fig. 7i and j) and BCAA (Figures S13e-S13g) and inhibited mTOR signaling pathway (Fig. 7i and j) in the heart of T1D mice, but not in FGF21-knockdown T1D mice. Collectively, our results reveal that hepatic FGF21 plays an important role in the protective effect of the gut microbiota on T1D-associated cardiac dysfunction.

### Zbtb7c mediates the inhibitory effect of FGF21 on cardiac LAT1 expression

**Fig. 8:**
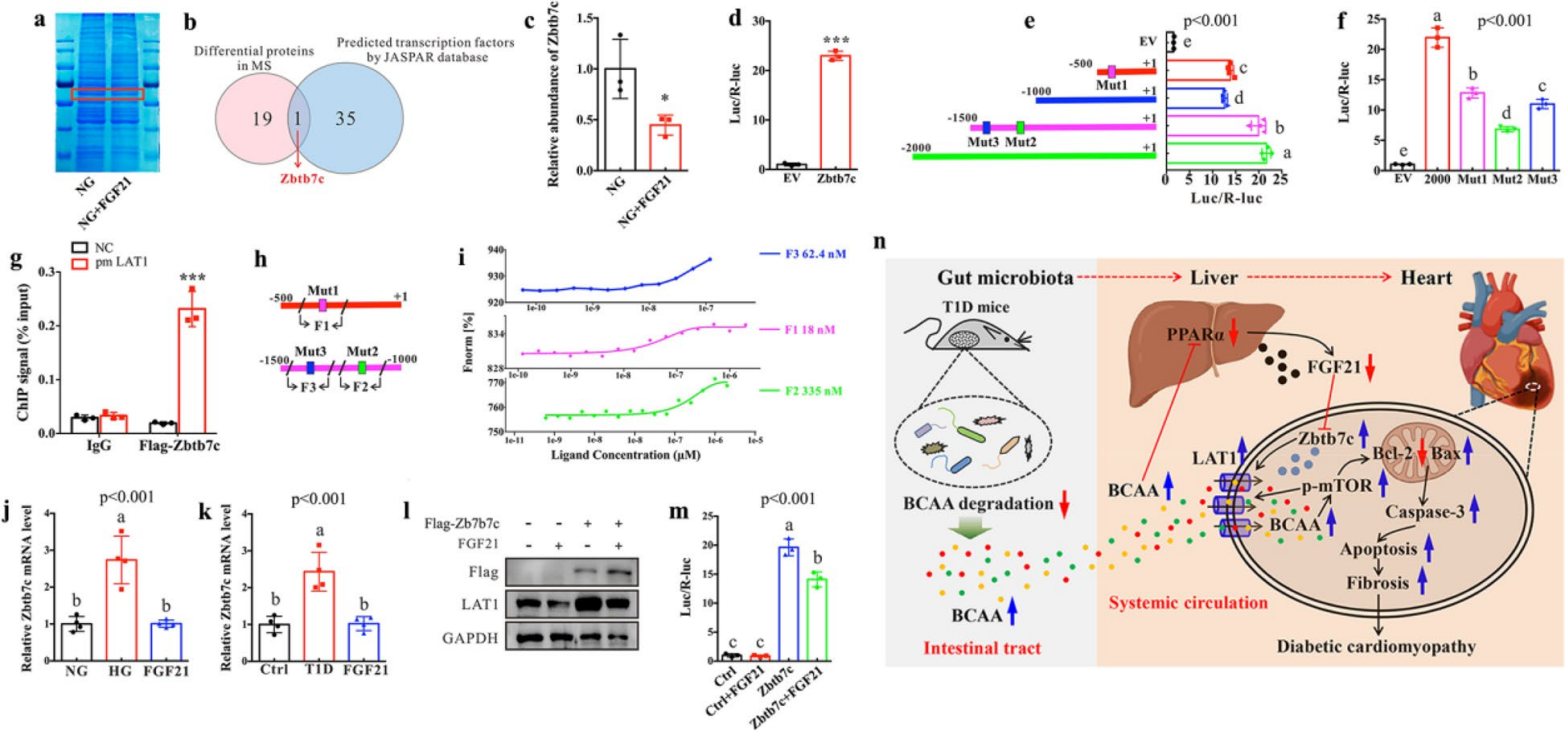
Zbtb7c serves as a key transcription factor for inhibition of LAT1 by FGF21. **a** Proteomics analysis: proteins were separated on SDS-PAGE, stained with Feto SDS-PAGE buffer and the target gel slices were excised and extracted for MS analysis. **b** Venn diagram showing one common protein that were significantly altered in H9c2 cells after FGF21 treatment and also predicted as transcription factors by JASPAR database. **c** The relative abundance of Zbtb7c protein in H9c2 cells after FGF21 treatment. **d** Luciferase reporter assay. HEK293 cells were transfected with Zbtb7c plasmid and full-length LAT1 promoter or pGL3-basic vector (EV), and the relative luciferase values were normalized to pGL3-basic vector. **e** the 2 kb promoter was fragmented into three segments (-500, -1000 and -1500 nucleotide sites), and the luciferase reporter assay showed that -500 to + 1 and -1000 to -1500 nucleotide sites were the major binding sequence of LAT1 for Zbtb7c-dependent activation. **f** The relative luciferase activity ofHEK293 cells after mutations at the binding sites of -500 to + 1 (Mut1) and -1000 to -1500(Mut2 and Mut3). **g** Chromatin immunoprecipitation (ChIP) assay. H9c2 cells were transfected with Zbtb7c or empty vector (NC) and subjected to ChIP analysis with Flag antibody. An IgG isotype control was used as negative control. **h** Cy5 labeled DNA fragments containing the binding sites of Mut1, Mut2 and Mut3. **i** Microscale thermophoresis (MST) assay was performed to determine the binding affinity of ZBTB7C and DNA fragments of LAT1 promoter region. X-axis is molar protein concentration and Y-axis is normalized fluorescence. Dissociation contants (Kd) values of Zbtb7c towards fragment 1 (F1), fragment 2 (F2) and fragment 3 (F3) of LAT1 promoter region are listed, respectively. **j** Relative Zbtb7c mRNA levels of H9c2 cells cultured under normal glucose (NG, 5.5 mM) and high glucose (HG, 33 mM) conditions and pretreated with FGF21 under the HG condition (FGF21). **k** Relative Zbtb7c mRNA levels in the heart of normal control (Ctrl), type 1 diabetic (T1D) and FGF21-treated T1D mice. **l** The expression level of LAT1 in H9c2 cells transfected with Zbtb7c plasmid or empty vector with and without pretreatment with FGF21. GAPDH was used as a loading control. **m** The relative luciferase activity of HEK293 cells transfected with Zbtb7c plasmid and full-length LAT1 promoter or pGL3-basic vector (EV) with and without pretreatment with FGF21. **n** The gut microbiota-derived metabolite BCAA mediated the microbiota-liver-heart crosstalk and affected DCM via FGF21

To further elucidate the potential mechanisms underlying the regulating effect of FGF21 on LAT1 expression, a comparative proteomics analysis was performed to examine FGF21-induced alterations of protein profile in H9c2 cells. A total of 20 differential proteins were identified in FGF21-treated H9c2 cells (Fig. 8a and b). In addition, we predicted 36 transcription factors in the promoter of LAT1 based on the JASPAR database [39]. Among these differential proteins, zinc-finger and BTB domain-containing 7C (Zbtb7c) was identified as a possible transcription factors of the LAT1 promoter related to FGF21 (Fig. 8b). Moreover, we observed that the relative abundance of Zbtb7c was significantly reduced in H9c2 cells after FGF21 treatment (Fig. 8c). Zbtb7c, as a member of the Zbtb transcription factor family, has been proved to modulate cell growth and proliferation [40]. Recent studies also reported that Zbtb7c has the ability to regulate glucose-lipid metabolism [41, 42]. To investigate whether Zbtb7c directly interacts with the LAT1 promoter element, we cloned the 2 kb LAT1 promoter into a luciferase reporter plasmid. Next, the 2 kb promoter reporter was co-transfected with empty vector (EV) or Zbtb7c plasmids into HEK293 cells and the relative light units (RLU) showed a robust and positive response induced by Zbtb7c, as shown in Fig. 8d. Next, the 2 kb promoter was fragmented into three segments including -500, -1000 and -1500 nucleotide sites, and the luciferase reporter results reveal that -500 to + 1 and -1000 to -1500 nucleotide sites were the major binding sequence of LAT1 for Zbtb7c-dependent activation (Fig. 8e). To further confirm the potential site of the LAT1 promoter binding to Zbtb7c, mutant LAT1 promoter was generated and co-transfected with Zbtb7c vector. Intriguingly, the alteration of individual mutation site reduced the Zbtb7c-dependent transactivation (Fig. 8f). As can be seen from Fig. 8g, the chromatin immunoprecipitation (ChIP) assay confirmed that Zbtb7c can directly interact with the LAT1 promoters in H9c2 cells. Additionally, the microscale thermophoresis (MST) assay was performed to determine the binding affinity between Zbtb7c and LAT1 (Fig. 8h), and the results show that Zbtb7c exhibited a strong binding affinities toward Mut1 (18 nM), Mut2 (335 nM) and Mut3 (62.4 nM) of LAT1 nucleotide sites (Fig. 8i). Furthermore, we further examined whether Zbtb7c is a key target transcription factor involved in the regulation of LAT1 by FGF21 in myocardial cells. We found that the mRNA level of Zbtb7c was significantly increased in both H9C2 cells under the HG condition (Fig. 8j) and the heart tissue of T1D mice (Fig. 8k) but significantly decreased after FGF21 treatment. To verify Zbtb7c involved in FGF21-induced regulation of LAT1, we detected the protein level of LAT1 in H9c2 cells with both Zbtb7c overexpression and FGF21 treatment. The results show that transfection with Zbtb7c significantly increased the protein expression level of LAT1 in H9c2 cells, but this increase was markedly blocked after FGF21 treatment (Fig. 8l). In addition, the luciferase reporter assay shows that Zbtb7c significantly enhanced the LAT1 promoter activity, but FGF21 treatment reduced its activity (Fig. 8m), suggesting that Zbtb7c serves as a key target transcription factor for the regulating effect of FGF21 on LAT1 expression.

## Discussion

Herein we reported that the gut microbiota-derived metabolite BCAA mediated the microbiota-liver-heart crosstalk and affected DCM via FGF21 (Fig. 8n). T1D mice exhibited a lower BCAA degradation ability of the gut microbiota, resulting in an increased BCAA intake from the gut to the circulation. Excess circulating BCAA reduced hepatic FGF21 production by inhibiting PPARα signaling pathway and thereby led to an increased expression level of cardiac LAT1 via transcription factor Zbtb7c. High LAT1 increased the levels of BCAA in the heart, and excess cardiac BCAA activated mTOR signaling pathway, caused mitochondrial injury and myocardial cell apoptosis, and contributed to cardiac fibrosis and dysfunction in T1D mice. Moreover, we found that FMT from healthy mice alleviated cardiac dysfunction in T1D mice, but this effect can be abolished by FGF21 knockdown.

BCAA, including leucine, isoleucine and valine, serve as essential nutrients and also signaling molecules for cellular growth and development [43]. BCAA affect multiple biological functions by targeting the mTOR signaling pathway, such as autophagy [44], insulin signaling [45], fatty acid metabolism [46], inflammation [47] and mitochondrial function [48]. Thus, BCAA have been linked to the onset and progression of diabetes [49], obesity [50], cardiovascular disease [22] and cancer [51]. Moreover, abnormal BCAA metabolism has also been regarded as a real culpritin heart diseases rather than an epiphenomenon [18]. In the current study, using an untargeted metabolomics analysis, higher BCAA levels and lower ATP level were identified as major metabolic characteristics in both diabetic heart and HG-induced myocardial cells. Furthermore, we verified that excess cardiac BCAA induced mitochondrial damage and myocardial cell apoptosis by activating mTOR signaling pathway and then caused cardiac fibrosis and dysfunction in T1D mice. Of note, herein we revealed that BCAA can increase the level of cardiac LAT1 as an important BCAA transporter [27] via the mTOR signaling pathway, resulting in further increased BCAA level in the heart. Inhibiting LAT1 can break this vicious circle and thereby protect cardiac health and functions in T1D mice, suggesting LAT1 as a possible target for treating DCM.

Elevated circulating BCAA levels have been detected in both types of diabetes [52–55]. In the present study, we revealed that increased circulating BCAA levels might be attributed to shifts in the gut microbial functions in T1D mice, especially reduced BCAA degradation ability, and FMT experiment further confirmed the vital role of the gut microbiota on host BCAA levels. Microbial modification by FMT has been used to treat recurrent *Clostridioides difficile* infection [56] and is also being explored for other diseases, such as ulcerative colitis [57], Alzheimer’s disease [58], myocarditis [59], colorectal cancer [60], metabolic syndrome [61] and diabetes [62]. Here we found that FMT effectively improved cardiac dysfunction in T1D mice through reshaping the BCAA degradation ability of the gut microbiota and suppressing LAT1-driven increase in BCAA.

Another interesting finding of this study is that the gut microbiota-derived BCAA decreased hepatic FGF21 production by inhibiting PPARα signaling pathway. In fact, the association between the gut microbiota and FGF21 has been proposed, for example, dietary protein restriction can increase FGF21 level in the liver of mice and this effect is mediated by the gut microbiota [63]. Kundu et al. reported that young germ-free mice after FMT from old mice exhibited increased hepatic FGF21 level [64]. In addition, of note, FGF21 has been shown to protect cardiomyopathy by inflammatory pathway [65], oxidative stress [66], AMP-activated protein kinase pathway [67] and lipid-lowering effect [68]. Herein we shed light on a novel protective mechanism of FGF21 on DCM by suppressing LAT1-driven increase in BCAA. This finding may also indicate FGF21 as a potential drug for other LAT1-related diseases. Moreover, in this study, we first discovered that a transcription factor Zbtb7c plays an essential role in the inhibition of cardiac LAT1 expression through FGF21. Zbtb7c has been reported as an important transcription factor for glucose-lipid metabolism [41, 42], tumorigenesis [69] and Mmp gene transcription [40]. We revealed that Zbtb7c is a key transcription factor of the LAT1 promoter but can be inhibited by FGF21. Additionally, FGF21 knockdown can abolish the protective effect of FMT on DCM in T1D mice, suggesting that FGF21 plays an essential role in the BCAA mediated microbiota-liver-heart crosstalk.

In conclusion, we provide evidence for the involvement of FGF21 in the BCAA mediated microbiota-liver-heart crosstalk during the development of DCM. An impaired crosstalk between the gut microbiota, liver and heart contributed to mitochondrial damage and fibrosis in the T1D heart, leading to cardiac dysfunction. Our study may promote research into improving communication between the gut microbiota and other organs for the treatment of diabetic heart diseases. Further studies are encouraged to explore whether targeting the microbiota-liver-heart axis can be translated into an effective treatment of DCM in clinical practice. However, there are still some limitations in this study that require further exploration: (1) Further clarification is needed on the effects of other amino acids beyond BCAA on cardiomyocyte apoptosis and myocardial fibrosis; (2) It remains unknown whether the pathophysiological mechanisms of T1D cardiomyopathy proposed herein also exist in T2D; (3) How to precisely modify the microbiota-liver-heart axis for cardiac health remains open and merits investigation.

## Materials and methods

### Animals

Six-week old male C57BL/6 mice (body weight = 20 ± 5 g) were purchased from the Vital River Laboratory Animal Technology (Beijing, China), and housed in the specific-pathogen-free (SPF) colony under a constant condition (temperature, 22 ± 2℃; humidity, 55 ± 5%) with a 12/12 h light/dark cycle at the Laboratory Animal Center of Wenzhou Medical University (Wenzhou, China). All mice were given free access to standard mouse chow and water, and acclimatized for 1 week prior to experiment. Mouse chow and drinking water were prepared by irradiation and steam autoclave sterilizations, respectively. This study was carried out according to the Guide for the Care and Use of Laboratory Animals and approved by the Institutional Animal Care and Use Committee of WMU (ID: xmsq2021-0714).

### Streptozotocin-induced type 1 diabetic mouse model

After a 1-week acclimation, all mice were weighted and randomly assigned into two groups, normal control (Ctrl) and diabetic groups. Streptozotocin (STZ) has been commonly used to develop an animal model of type 1 diabetes (T1D), since STZ can targetedly cause pancreatic β-cell destruction [70]. To develop a mouse model of type 1 diabetes (T1D), mice after a 12-h fasting were treated with intraperitoneal injection of STZ (Sigma-Aldrich) solution in citrate buffer (pH = 4.5) at a dose of 50 mg/kg body weight for 5 consecutive days. The Ctrl mice were given by intraperitoneal injection with the same volume of citrate buffer. Blood glucose level was measured using a handheld glucometer (ACCU-CHEK Active, Mannheim, Germany) after 3 days of STZ injection, and T1D mice were selected when blood glucose level > 11.1 mmol/L. In addition, body weight, blood glucose level, food intake and water intake were monitored once a week throughout the experimental period.

### Fecal microbiota transplantation (FMT)

After 8 weeks of T1D development, T1D mice were randomly assigned into T1D and FMT groups. T1D mice in the FMT group were treated with an antibiotic cocktail of four oral antibiotics (vancomycin, 0.5 g/L; metronidazole, 1 g/L; ciprofloxacin, 0.2 g/L; amphotericin B, 0.01 g/L) to deplete their indigenous intestinal bacteria for 5 days. In addition, a group of normal Ctrl mice were used as donors for FMT. Fresh faecal pellets were collected from Ctrl donor mice and dissolved in sterile PBS (1:7.5, w/v), and the mixture were vortexed for 1 min and centrifuged at 1,000 g for 5 min. Each recipient T1D mouse was immediately gavaged with 100 μL of the faecal bacterial supernatant once a day for 2 weeks. Moreover, another group of T1D mice were given the same volume of sterile PBS buffer by gavage as a control.

### Echocardiography

After an 8-week T1D development, mice were anesthetized using inhaled isoflurane at 2–3% in a box and maintained with inhaled isoflurane (0.5–1%) by a nose cone, and subsequently their cardiac systolic and diastolic functions were measured by using transthoracic echocardiography (Vevo 3100, Visual Sonics, Canada) with a 15-MHz linear array ultrasound transducer. In this study, left ventricular fractional shortening (FS%), left ventricular ejection fraction (EF%) and left ventricular internal diameter at end-systole (LVIDs) were calculated to indicate cardiac functions of mice.

### Cell culture and treatment

H9c2 rat cardiomyocytes were purchased from Shanghai Institute of Biochemistry and Cell Biology (Shanghai, China) and cultured in the Dulbecco's modified Eagle's medium containing 1 g/L glucose, 10% fetal bovine serum (Gibco, Eggenstein, Germany) and 1% penicillin–streptomycin (Invitrogen, CA, USA). H9c2 cells were cultured under a humidified atmosphere condition (5% CO_2_ and 95% air) at 37 °C. To examine the effect of high glucose on myocardial cells, H9c2 cells were exposed to either normal glucose (NG, 5.5 mM) or high glucose (HG, 33 mM) for 48 h. To investigate the effects of BCAA on myocardial cells, H9c2 cells were cultured under the NG media supplemented with leucine (Leu, 10 mM), isoleucine (Ile, 5 mM) and valine (Val, 5 mM) for 48 h, respectively. Amino acid compositions of H9c2 cell culture media were listed in Table S4. In addition, H9c2 cells were cultured under the HG media supplemented with Rapamycin (50 nM) for 48 h to study the role of mTOR signaling pathway.

To achieve overexpression of LAT1, the coding DNA sequence of LAT1 was amplified and cloned into expression vector pcDNA3.1 (Invitrogen, CA, USA). To achieve knockdown of LAT1, LAT1-specific shRNA was designed and synthesized by GenePharma Company (Shanghai, China). The transfection was conducted by using Lipofectamine 3000 reagent (Invitrogen, CA, USA) according to the manufacturers’ instruction. Stable clones of LAT1-overexpressed and LAT1-knockdown H9c2 cells were selected with G418 and purimycin, respectively.

### FGF21 treatment

T1D mice were intraperitoneally injected with FGF21 at a dose of 1 mg/kg body weight every other day for 8 weeks after STZ injection. For JPH203 treatment, T1D mice were intraperitoneally injected with JPH203 (SelleckChem, Houston, TX, USA) at a dose of 10 mg/kg body weight every other day for 8 weeks. Meanwhile, T1D mice in the corresponding control group were given the same volume of saline. For in vitro experiment, H9c2 cells were pretreated with FGF21 (100 ng/mL) for 2 h or treated with JPH203 (10 μM), and then cultured under the HG condition (33 mM glucose) for 48 h.

### BCAA treatment

Mice after one week acclimation were given BCAA in their drinking water (10 mM leucine, 5 mM isoleucine and 5 mM valine) daily for 12 weeks and then subjected to cardiac function testing and sample analysis. For in vitro experiment, the hepatocyte cell line AML-12cells were treated with BCAA (10 mM leucine, 5 mM isoleucine and 5 mM valine) under the NG condition (5.5 mM glucose) for 48 h and then harvested for analysis.

### Adeno-associated virus (AAV) treatment

In this study, adeno-associated virus (AAV) vectors are used for heart-specific LAT1 overexpression and liver-specific FGF21 knockdown in mice. AAV9-LAT1 and AAV8-shFGF21 were constructed and purchased from OBiO Technology (Shanghai, China). To obtain heart-specific LAT1 overexpression, 100 μL of AAV9-LAT1 vector solution (2x10^11^ viral titer) was injected into T1D mice through tail vein at 0 and 4 weeks after STZ treatment, while T1D mice in the negative control (NC) group were injected with the same volume of AAV9-NC vector solution (empty vector). For liver-specific FGF21 knockdown, T1D mice were injected with 100 μL of AAV8-shFGF21 solution (1x10^11^ viral titer) from tail vein at 4 and 8 weeks after STZ treatment, and T1D mice in the NC group were injected with the same volume of AAV8-NC vector solution.

### Sample collection

Prior to sacrifice, faecal pellets were freshly collected from mice in a metabolic cage and immediately stored at -80 ℃ until use. Mice were anaesthetized using isoflurane and sacrificed by rapid decapitation. Blood samples were collected from the orbit, stood on ice for 15 min and centrifuged at 3,000 g at 4 ℃ for 15 min to separate serum samples, and stored at -80 ℃ until analysis. Moreover, heart and liver tissues were also harvested, rapidly frozen in liquid nitrogen and kept in -80 ℃ until use.

### Metabolite extraction

Metabolite extraction from heart tissue was carried out by using the MCW method as described by Zheng et al. [71]. In brief, 0.1 g of the tissue was weighed and homogenized with 4 mL/g of ice-cold methanol and 0.85 mL/g of ice-cold water. Then, the mixture was added with 2 mL/g of ice-cold chloroform and water. After vortexing for 30 s, the mixture was kept on ice for 15 min and centrifuged at 12,000 g for 15 min at 4 °C. The supernatant was lyophilized and stored at -80 °C for further analysis. To extract serum metabolites, 200 μL of serum was thawed and added to 250 μL of PBS (0.2 mM Na_2_HPO_4_/NaH_2_PO_4_, pH = 7.4) and 50 μL of D_2_O (99.5%, Isotope Laboratory, CA, USA). The diluted serum sample was vortexed for 30 s, centrifuged at 12,000 g for 15 min at 4 °C, and then 500 μL of supernatant was collected for LC–MS analysis. For faecal metabolite analysis, 0.1 g of faecal pellet was weighed and homogenized thoroughly with 0.5 mL of PBS in a 1.5 mL centrifuge tube for 5 min. The mixture was centrifuged at 5,000 g for 15 min at 4 °C, and 400 μL of the supernatant was mixed with 100 μL of D_2_O containing 0.05% of sodium trimethylsilyl propionate-d_4_ (TSP, 0.42 mM) for NMR analysis.

Intracellular and extracellular metabolites were extracted according to a previously published method [72]. Briefly, 1 mL of culture medium was added to 3 mL of ice-cold methanol/chloroform (2:1, v/v) mixture and transferred into a 15 mL centrifuge tube. Subsequently, 1 mL of ice-cold chloroform was added, vortexed vigorously for 30 s, and centrifuged at 10,000 g for 15 min at 4 °C. The supernatant was lyophilized and stored at -80 °C for subsequent analysis. For intracellular metabolites, the cells were harvested and washed three times with ice-cold PBS. The cells pellets were then suspended in 500 μL of ice-cold methanol/chloroform (2:1, v/v) mixture in a 1.5 mL centrifuge tube and vortexed vigorously for 30 s. The mixture was incubated at 4 °C for 10 min, added with 200 μL of ice-cold chloroform/water (1:1, v/v), and ultrasonicated on ice for 10 min. Then, the mixture was centrifuged at 12,000 g for 20 min at 4 °C and the supernatant was lyophilized and kept at -80 °C for metabolomics analysis.

### NMR-based metabolomics analysis

^1^H NMR spectra were acquired by using Bruker AVANCE III 600 MHz spectrometer (BrukerBioSpin, Rheinstetten, Germany) equipped with a 5-mm TXI probe at 298 K.

The lyophilized powders obtained from heart, cellular and medium extracts were dissolved in 500 μL of D_2_O containing TSP (0.42 mM) and transferred into a 5 mm NMR tube for metabolomics analysis. A standard single-pulse sequence with water signal presaturation (ZGPR) was used to record their metabolic profiles, and the main parameters were set as follows: scans = 256; spectral width = 12,000 Hz; data points = 256 K; relaxation delay = 4 s; acquisition time = 2.66 s/scan.

All NMR spectra were manually preprocessed for phase/baseline corrections and referenced to TSP peak at 0 ppm using Topspin software 3.0 (BrukerBiospin, Germany). The “icoshift” procedure was employed to align all NMR spectra of each type of sample with MATLAB software (R2018b, The Mathworks Inc., MA, USA). Afterward, the NMR spectral region from 0.0 to 9.9 ppm excluding residual water signals from 4.7 to 5.2 ppm was subdivided into 0.01 ppm interval for multivariate data analysis. Metabolite signals were identified on the basis of the Chenomx NMR suite 7.0 (Chenomx Inc., Edmonton, Canada) and HMDB 4.0 [73]. The [13] C-^1^H heteronuclear single quantum coherence (HSQC) experiment was used to further confirm uncertain identifications. Finally, the relative level of metabolite was calculated on the basis of its peak area by reference to TSP peak area and concentration.

### Measurement of BCAA by UPLC-MS

The concentrations of BCAA were measured by using an API 6500 Q-TRAP (AB SCIEX, Foster City, CA, USA) mass spectrometer with a SHIMADZU CBM-30A Lite LC system (Shimadzu Corporation, Kyoto, Japan) under positive (ESI +) model. Prior to LC–MS analysis, the lyophilized extract was redissolved in deionized water and added with 100 μL of 0.1% formic acid. Then the sample was vigorously vortexed for 30 s and centrifuged at 12,000 g for 20 min, and the supernatant was used for analysis. The LC separation was carried out on a Waters Acquity BEH C18 column (2.1 × 100 mm, 1.7 μm) (Waters Corporation, Milford, USA) using a gradient of solvent A (0.1% formic acid in water) and solvent B (0.1% formic acid in acetonitrile) at a flow rate of 0.3 mL/min. The LC gradient was set as follows: 0 min, 98–2% B; 2.5 min, 98–2% B; 3 min, 90–10% B; 4 min, 5–95% B; 5 min, 5–95% B; 5.5 min, 98–2% B; 7.5 min, 98–2% B. Auto-sampler temperature was 5 °C, and injection volume was 1 μL. The ion source parameters mainly included: curtain gas (CUR) = 28 psi; ion spray voltage (IS) = 5500 V; temperature (TEMP) = 500 °C; ion source gas 1 (GS1) = 55 psi; GS2 = 55 psi; collision gas (CAD) = medium. Data was acquired in multiple reaction monitoring (MRM) with transitions as follows: m/z 118.0 to 72.1 for valine and m/z 132.1 to 86.1 for leucine and isoleucine. Authentic standard BCAA were used to perform standard curves to quantify the concentrations of BCAA. Data were analyzed by using AB Sciex MultiQuant software (v.3.0.3, AB SCIEX, Foster City, CA, USA).

### Transcriptomics analysis

Total RNA was extracted from liver tissue by using Trizol reagents (Invitrogen, CA, USA) and the RNA concentration was measured with NanoDrop 2000 (Thermo Fisher Scientific, Wilmington, USA). The RNA integrity was determined by the RNA Nano 6000 Assay Kit of the Bioanalyzer 2100 system (Agilent Technologies, USA). Transcriptome sequencing libraries were generated with NEBNext Ultra TM RNA Library Prep Kit for Illumina (NEB, USA) according to the manufacturer’s protocol. The index-coded samples were then clustered on a cBot Cluster Generation System with a TruSeq PE Cluster Kit v4-cBot-HS (Illumina). Transcriptome data were obtained by the Illumina NovaSeq platform and 150 bp paired-end reads were obtained after processing. Clean reads were generated via removing adapter, poly-N and low-quality reads from raw data. Finally, KEGG pathway database was used to achieve the functional annotation of the transcripts.

### Proteomics analysis

H9c2 cells were treated with or without FGF21 (100 ng/mL) for 48 h and the cells pellets were harvested for protein extraction. Proteins were separated on SDS-PAGE, stained with Feto SDS-PAGE buffer (absci, WA, USA) and the target gel slices were excised. The gel slice was distained with a solution of 50 mM ammonium bicarbonate and 50% acetonitrile. Disulfide bonds were reduced with DTT, alkylated with iodoacetamide and digested with 10 ng/μL trypsin. The Next day digested peptides were extracted with 50% acetonitrile (ACN) containing 0.5% formic acid (FA), lyophilizedbya vacuum centrifuge. Finally, peptides were dissolved in 20% ACN containing 0.5% FA and analyzed by using a Orbitrap Fusion Lumos System (Thermo Fisher Scientific, MA, USA) with a C18 7.5 μm × 100 mm trap column.

### Histological analysis

In this study, mice (*n* = 3) were anaesthetized with isoflurane and sacrificed by normal saline perfusion. The heart and liver tissues were rapidly collected and fixed with 4% paraformaldehyde in PBS (0.1 M, pH = 7.5). The tissue sample was dehydrated with a graded series of ethanol, embedded in paraffin and sectioned into 5 μm slices using a slicing machine (Leica, Germany). To assess the degree of myocardial fibrosis, the cardiac section was dewaxed with xylene, rehydrated in gradient alcohol, and stained with Masson’s trichrome (Beyotime, China). For immunohistochemical analysis, the tissue section after deparaffinization and rehydration was incubated in 3% H_2_O_2_ for 10 min to quench endogenous peroxidase, placed in citrate buffer (pH = 6.5, 1:20, v/v) within a decloaking chamber for 5 min and cooled at room temperature. The tissue section was washed three times with PBS and blocked in 5% BSA for 1 h, followed by incubation with primary antibodies directed against L-type amino acids transporter (LAT1, cat# sc-374232, Santa Cruz, CA, USA) or fibroblast growth factor 21 (FGF21, cat# ab171941, Abcam, Cambridge, UK) overnight at 4 °C. Then, the tissue section was washed and incubated with anti-mouse and anti-rabbit antibodies for 1 h, respectively, and washed three times with PBS. The image was captured using light microscopy (Nikon, Japan) and the corresponding quantitative analysis was performed by Image J software (v1.47, National Institutes of Health, Bethesda, MD, USA).

For immunofluorescence analysis, the cardiac section was dewaxed, rehydrated and incubated with wheat germ agglutinin (WGA, cat# GTX01502, GeneTex, CA, USA) overnight in the dark at 4 °C to visualize cardiomyocyte membrane. To measure the number of apoptotic cells, H9c2 cells were fixed with 4% paraformaldehyde for 15 min and stained with Hoechst 33342 (cat# C1025, Beyotime, China) in the dark for 5 min. Finally, the tissue and cell sections were washed three times with PBS. The image was captured using confocal microscope (A1R-SIMSTORM, Nikon, Japan) and the corresponding quantitative analysis was carried out by Image J software.

### Flow cytometry analysis

The percentage of apoptotic cells was detected by an Annexin V-FITC apoptosis detection kit for flow cytometry according to the manufacturer’s instruction (BD, Biosciences, CA, USA). In brief, H9c2 cells (1 × 10^6^ cells/well) were harvested and washed twice with cold PBS, and then suspended with 500 μL of binding buffer. Subsequently, the cells were incubated with 5 μL Annexin V and 1 μL PI solution for 15 min in the dark. Data were acquired on flow cytometry (FACS Calibur, BD, Biosciences, CA, USA) and analyzed by Cell Quest software (BD, Biosciences, CA, USA).

### Mitochondrial membrane potential measurement

Mitochondrial membrane potential was determined by JC-1 fluorescent probe according to the manufacturer’s instruction (cat# C2006, Beyotime, Nantong, China). H9c2 cells were cultured on a confocal petri dish at a density of 1 × 10^6^ cells/well. After experimental treatment, the cells were washed three times with PBS and incubated with 5 μg/mL Mitoprobe JC-1 in the dark for 30 min. After washing twice with PBS, the cells were cultured in 1 mL of incubation buffer and analyzed immediately under a fluorescent microscope (Olympus, Tokyo, Japan). Green and red fluorescence represent the monomeric and aggregation forms of JC-1, and indicate mitochondrial membrane depolarization and actively respiring mitochondria, respectively. The relative fluorescence level was measured using Image J software (v1.47, National Institutes of Health, Bethesda, MD, USA).

### Mitochondrial respiration assay

Cellular mitochondrial respiration and glycolysis were measured on Seahorse XF96 analyzer (Agilent Seahorse Bioscience, CA, USA) with Seahorse XF Cell Mito Stress Test Kit (Agilent, cat# 103,344–100) and Glycolysis Rate Assay Kit (Agilent, cat# 103,010–100), respectively. In brief, H9c2 cells (1 × 10^5^ cells/well) after experimental treatment were seeded in Seahorse 96-well assay plates and the culture media were changed to 200 μL of the assay buffer before the test. Oligomycin (1 μM), FCCP (1 μM) and rotenone/antimycin (Rote/AA, 0.5 μM) were injected sequentially into the culture media to evaluate oxygen consumption rate (OCR), while Rote/AA (0.5 μM) and 2-deoxy-D-glucose (2-DG, 1 μM) were added into the culture media to measure extracellular acidification rate (ECAR). Finally, OCR and ECAR values were calculated and normalized to the protein content of each well using Seahorse Wave software (Agilent Seahorse Bioscience, CA, USA).

### Metagenomic sequencing analysis

Faecal pellets were freshly collected from mice in sterile containers and immediately stored at -80 °C until analysis. The microbial genomic DNA was extracted by using the stool DNA isolation kit (TianGen, Beijing, China) according to the manufacturer’s instruction. For metagenomic sequencing, approximately 1 μg of the microbial DNA for each sample was broken into 350 bp reads via a sonication method, and a sequencing library was generated with NEBNext® Ultra™ DNA Library Prep Kit for Illumina (NEB, USA) and index codes were employed to attribute sequences to each sample. Subsequently, the DNA fragments were end-polished, A-tailed and ligated with the full-length adaptor for Illumina sequencing and amplified by PCR. The PCR products were purified by the AMPure XP system (Beckman) and the index-coded samples was clustered on a cBot Cluster Generation System with TruSeq PE Cluster Kit v4-cBot-HS (Illumina) according to the manufacturer’s instruction. Finally, the library preparations were sequenced on an Illumina HiSeq2500 PE150 sequencer at Novogene (Beijing, China).

The sequencing data were subjected to bioinformatics analysis. Briefly, low-quality reads were filtered by the Readfq (v8.0) to acquire clean data and then assembled to generate a number of scaffolds using SOAPdenovo software (v2.04). The assembled scaffolds were used to predict open reading frames (ORFs) in MetaGeneMark (v2.10), and the redundant genes were removed with CD-HIT software (v4.5.8). The unigenes sequence files were analyzed by DIAMOND (v0.9.9.110) on the basis of NR database at NCBI with the Basic Local Alignment Search Tool (BLAST). The lowest common ancestor (LCA) algorithm was used to assign ORFs alignments into taxonomic groups. The functional profile of KEGG orthology (KO) was predicted with PICRUSt software and then the predicted KO were categorized into KEGG pathways. The alpha-diversity of the gut microbiota was analyzed using QIIME software (v1.7.0) and nonmetric multidimensional scaling (NMDS) analysis was used to evaluate the beta-diversity of the gut microbiota.

### Measurement of malondialdehyde (MDA) and reduced glutathione (GSH)

Heart tissue was homogenized in 1 mL of PBS and centrifuged at 12,000 g for 20 min at 4 °C, and the supernatant was collected for subsequent analysis. The MDA level in the heart tissue was determined using the MDA assay kit (Beyotime Biotech, Nantong, China) based on thiobarbituric acid (TBA) method. In brief, the extract supernatant was added with TBA and incubated at 90 °C for 40 min, and centrifuged at 4,000 g for 10 min. Then the supernatant was collected and measured at 532 nm with a microplate reader (Bio-Tek Instruments Inc., VT, USA). The GSH level in the heart tissue was determined using the GSH assay kit (Beyotime Biotech, Nantong, China) based on the chemical conjugation of GSH with 5,5’-Dithiobis (DTNB). Briefly, the extract supernatant was added with masking reagent binding GSH and incubated in buffer at 37 °C for 30 min. The levels of total GSH and oxidized GSH (GSSG) were assessed at 405 nm with a microplate reader (Bio-Tek Instruments Inc., VT, USA) at 30 s and 10.5 min after the reaction at room temperature. According to the manufacturer’s instruction, the level of GSH was calculated as total GSH-GSSG × 2.

### Endogenous FGF21 assay

The level of serum FGF21 was assessed in a 96-well microplate by a mouse FGF21 ELISA kit (cat# MFG028, Immuno Diagnostics, Saritavihar, Delhi, India) according to the manufacturer’s instruction and measured at 405 nm with a microplate reader (Bio-Tek Instruments Inc., VT, USA).

### Western blot analysis

Total protein from cell and tissue samples were extracted with lysis buffer containing phosphatase inhibitors (Beyotime, China) and its concentration was determined using Bradford assay kit (Beyotime, China). Cellular or tissue lysates were separated on 10% SDS-PAGE gel at 120 V and transferred to a PVDF membrane (Millipore, Billeria). The membranes were blocked with 5% non-fat milk for 2 h at room temperature, and incubated with primary antibodies overnight at 4 °C. After washing five times with TBST, secondary antibodies were incubated for 1 h at room temperature and the protein bands was detected using a chemiluminescence (ECL) kit (Millipore, Billeria). The expression level of protein was normalized to respective protein controls and quantified using Image J software. Primary antibodies used in this study included: GAPDH (1:1000, cat#AF0006, Beyotime), LAT1 (1:1000, cat# sc-374232, Santa Cruz), Collagen-1 (1:1000, cat# ab34710, Abcam), Fibronectin (FN, 1:1000, cat# ab2413, Abcam), Bax (1:1000, cat# 60267, Proteintech), Bcl-2 (1:1000, cat# 12789, Proteintech), Caspase-3 (1:1000, cat# 9665, CST), ANP (1:1000, cat# 27426, Proteintech), BNP (1:1000, cat# ab20984, Abcam), mTOR (1:1000, cat# 2971, CST), p-mTOR (1:1000, cat# 5536, CST) and FGF21 (1:1000, cat# ab171941, Abcam).

### Quantitative real-time PCR

Total RNA in cell, heart and liver tissues were extracted by using Trizol reagents (Invitrogen, CA, USA) and reverse-transcribed into complementary DNA (cDNA) through a PrimeScriptTM RT reagent kit (TaKaRa, RR037A, Tokyo, Japan) following the manufacturer’s instructions. The cDNA was subjected to quantitative mRNA analysis using a Light Cycler 480 PCR System (Roche, Basel, Switzerland) with AceQ Universal SYBR PCR Master Mix (cat# Q511-02, Vazyme, Nanjing, China). Then, the expression level of mRNA was quantified using the ΔΔCT method and GAPDH was used as a calibrator gene. All experiments were conducted at least in triplicates. Primer pairs used in this study were listed in Table S5.

### Luciferase reporter assay

The pcDNA3.1 or the Zbtb7c plasmid and LAT1-based firefly luciferase reporter plus pRL-TK were co-transfected with HEK293 cells for 48 h. After transfection, a dual-luciferase assay was performed using Luc-Pair Duo-Luciferase HS Assay Kit (GeneCopoeia, China) and measured using a SynergyNeo2 Microplate Reader (Biotek Instruments, Inc., Winooski, VT, USA). The ratio of firefly luciferase activity to Renilla luciferase activity was calculated as the relative luciferase activity.

### Chromatin immunoprecipitation (ChIP) assay

The ChIP assay was performed using a Simple ChIP® Enzymatic Chromatin IP Kit (#9002, CST, USA). Briefly, H9c2 cells were transfected with Zbtb7c or empty vector and cross-linked DNA by formaldehyde, and chromatin was cleaved to lengths of 150–500 bp by Micrococcal Nuclease and sonication. Chip was performed using 10 μg of antibodies against flag (GenScript, A00187) or Ig G (#2729, CST, USA) overnight at 4℃. The purified DNA was quantified by real-time PCR to amplify ZBTB7C-binding sites. The result was calculated as fold-enrichment relative to IgG. The primers used in this study are shown in Table S6.

### Microscale thermophoresis (MST) assay

The cDNA encoding Zbtb7c was cloned into the NdeI and EcoRI sites of the pET28b and the recombinant plasmid was transformed into *E. coli* strain BL21 (DE3). Recombinant protein expression was induced by isopropyl‐β‐D‐thiogalactopyranoside (IPTG) for 16 h at 16 °C in *E. coli*. The supernatant of cell culture containing secreted Zbtb7c was harvest and the purified of recombinant protein was conducted as previously reported [74]. Cy5 labeled DNA fragment was produced via chemical synthesis (3’-Cy5; GeneCopoeia). The fragments used herein were designed as follows: fragment 1 (F1**,** 5’-TAAATGGGGGTGGGCTG-3’);

fragment 2 (F2, 5’-TGGGTTGGTGGTATGCTT-3’);

fragment 3 (F3, 5’-CTGGGGACCCCCTTGGAT-3’).

The purified protein was serially diluted with 1 × PBST buffer and Cy5-labeled DNA was diluted in buffer with 10 mM Tris (pH 8.0), 200 mM NaCl and 0.05% Tween-20. Samples were loaded into Monolith™ Premium Capillaries and then microscale thermophoresis (MST) analysis was carried out by NanoTemper Monolith New Monolith (NanoTemper Technologies GmbH, Munich, Germany). The binding affinity was determined by fitting data from at least three independent experiments using NanoTemper analysis software.

### Statistical analysis

In this study, all mice and cells were randomly assigned to experimental procedures, and all analyses were conducted by masking group labels. Metabolomics data were Pareto-scaled and log-transformed, and orthogonal partial least squares discriminant analysis (OPLS-DA) was utilized to analyze the metabolic difference between two groups by SIMCA-P + software (v.12.0, Umetrics AB, Umeå, Sweden) and important metabolites were identified using S-plot. Beta-diversity of the gut microbiota was analyzed by nonmetric multidimensional scaling (NMDS) analysis using R software (v.2.15.3).

The difference between two groups and among multiple groups were determined by two-tailed unpaired student’s t test and one-way ANOVA in SPSS software (IBM SPSS statistics 22), respectively. Additionally, the difference between two groups with time was analyzed with a repeated measure ANOVA in SPSS software. The difference was considered as statistically significant when *p* < 0.05, and different lowercase letters indicate statistically significant differences.

### Supplementary Information

Supplementary Material 1: Table S1. Metabolic differences in heart tissue between normal control (Ctrl) and type 1 diabetic (T1D) mice. Table S2. Intracellular metabolic differences of H9C2 cells cultured under normal glucose (NG) and high glucose (HG) conditions. Table S3. Extracellular metabolic differences of H9C2 cells cultured under normal glucose (NG) and high glucose (HG) conditions. Table S4. Amino acid compositions of H9c2 cell culture media. Table S5. Primer pairs used in this study. Table S6. Primer pairs used for ChIP assay in this study. Figure S1. Diabetic cardiomyopathy occurs in streptozotocin (STZ)-induced type 1 diabetic mice. Figure S2. High glucose induces myocardial apoptosis. Figure S3. NMR-based metabolomics profiling. Figure S4. Faecal microbiota transplantation (FMT) partly corrects the gut microbiota in T1D mice. Figure S5. Faecal microbiota transplantation (FMT) reshapes the microbial function in T1D mice. Figure S6. Changes in BCAA catabolism enzymes both in vivo and in vitro. Figure S7. Changes in BCAA and their transporter LAT1 both in vivo and in vitro. Figure S8. Rapamycin alleviates mitochondrial damage and apoptosis of myocardial cells. Figure S9. Faecal microbiota transplantation (FMT) alleviates cardiac injure in T1D mice by reducing LAT1-driven increase in BCAAs and inhibiting mTOR pathway. Figure S10. Excess BCAA decreases FGF21 production both in vivo and in vitro. Figure S11. FGF21 alleviates mitochondrial damage and apoptosis of myocardial cells. Figure S12. FGF21 reduces BCAA in H9c2 cells under high glucose condition. Figure S13. The effect of AAV-mediated FGF21 knockdown on hepatic FGF21 production and cardiac BCAA levels in T1D mice with FMT.

## Data Availability

All data used in this study are present in the main text and supplementary materials. Omics data have been made publicly available in Figshare: Metabolomics (10.6084/m9.figshare.21523533.v1); Microbiome (10.6084/m9.figshare.21542388.v1); Transcriptomics (10.6084/m9.figshare.21529227.v1). Additional data and materials can also be requested from corresponding author.

## Abbreviations

ANP: Atrial natriuretic peptide
BCAA: Branched-chain amino acids
BNP: B-type natriuretic peptide
DCM: Diabetic cardiomyopathy
ECAR: Extracellular acidification rate
EF: Ejection fraction
FMT: Fecal microbiota transplantation
FN: Fibronectin
FS: Fractional shortening
GSH: Reduced glutathione
HG: High glucose
LAT1: L-type amino acid transporter 1
LVIDs: Left ventricular internal diameter at end-systole
MDA: Malondialdehyde
MMP: Mitochondrial membrane potential
NC: Normal control
NG: Normal glucose
OCR: Oxygen consumption rate
T1D: Type 1 diabetes

## References

[1] Parim B, Uddandrao VS, Saravanan G. Diabetic cardiomyopathy: molecular mechanisms, detrimental effects of conventional treatment, and beneficial effects of natural therapy. Heart Fail Rev. 2019;24(2):279–99. 30349977 10.1007/s10741-018-9749-1

[2] Tan Y, Zhang Z, Zheng C, Wintergerst KA, Keller BB, Cai L. Mechanisms of diabetic cardiomyopathy and potential therapeutic strategies: preclinical and clinical evidence. Nat Rev Cardiol. 2020;17(9):585–607. 32080423 10.1038/s41569-020-0339-2 PMC7849055

[3] Jia G, Hill MA, Sowers JR. Diabetic cardiomyopathy: an update of mechanisms contributing to this clinical entity. Circ Res. 2018;122(4):624–38. 29449364 10.1161/CIRCRESAHA.117.311586 PMC5819359

[4] Bugger H, Abel ED. Molecular mechanisms of diabetic cardiomyopathy. Diabetologia. 2014;57(4):660–71. 24477973 10.1007/s00125-014-3171-6 PMC3969857

[5] Brunvand L, Fugelseth D, Stensaeth KH, Dahl-Jørgensen K, Margeirsdottir HD. Early reduced myocardial diastolic function in children and adolescents with type 1 diabetes mellitus a population-based study. BMC Cardiovasc Disord. 2016;16(1):1–5. 27225446 10.1186/s12872-016-0288-1 PMC4881039

[6] Tang WW, Kitai T, Hazen SL. Gut microbiota in cardiovascular health and disease. Circ Res. 2017;120(7):1183–96. 28360349 10.1161/CIRCRESAHA.117.309715 PMC5390330

[7] Witkowski M, Weeks TL, Hazen SL. Gut Microbiota and Cardiovascular Disease. Circ Res. 2020;127(4):553–70. 32762536 10.1161/CIRCRESAHA.120.316242 PMC7416843

[8] Wang Z, Klipfell E, Bennett BJ, Koeth R, Levison BS, DuGar B, et al. Gut flora metabolism of phosphatidylcholine promotes cardiovascular disease. Nature. 2011;472(7341):57–63. 21475195 10.1038/nature09922 PMC3086762

[9] Schiattarella GG, Sannino A, Toscano E, Giugliano G, Gargiulo G, Franzone A, et al. Gut microbe-generated metabolite trimethylamine-N-oxide as cardiovascular risk biomarker: a systematic review and dose-response meta-analysis. Eur Heart J. 2017;38(39):2948–56. 29020409 10.1093/eurheartj/ehx342

[10] Heianza Y, Ma W, DiDonato JA, Sun Q, Rimm EB, Hu FB, et al. Long-term changes in gut microbial metabolite trimethylamine N-oxide and coronary heart disease risk. J Am Coll Cardiol. 2020;75(7):763–72. 32081286 10.1016/j.jacc.2019.11.060 PMC8140616

[11] Tang WW, Wang Z, Li XS, Fan Y, Wu Y, Hazen SL. Elevated gut microbiota dependent metabolite trimethylamine N-oxide (TMAO) is associated with subclinical myocardial dysfunction and necrosis in stable cardiac patients. Circulation. 2017;136:A17597.

[12] Marques FZ, Nelson E, Chu PY, Horlock D, Fiedler A, Ziemann M, et al. High-fiber diet and acetate supplementation change the gut microbiota and prevent the development of hypertension and heart failure in hypertensive mice. Circulation. 2017;135(10):964–77. 27927713 10.1161/CIRCULATIONAHA.116.024545

[13] Bartolomaeus H, Balogh A, Yakoub M, Homann S, Markó L, Höges S, et al. Short-chain fatty acid propionate protects from hypertensive cardiovascular damage. Circulation. 2019;139(11):1407–21. 30586752 10.1161/CIRCULATIONAHA.118.036652 PMC6416008

[14] Kaye DM, Shihata WA, Jama HA, Tsyganov K, Ziemann M, Kiriazis H, et al. Deficiency of prebiotic fiber and insufficient signaling through gut metabolite-sensing receptors leads to cardiovascular disease. Circulation. 2020;141(17):1393–403. 32093510 10.1161/CIRCULATIONAHA.119.043081

[15] Rainer PP, Primessnig U, Harenkamp S, Doleschal B, Wallner M, Fauler G, et al. Bile acids induce arrhythmias in human atrial myocardium-implications for altered serum bile acid composition in patients with atrial fibrillation. Heart. 2013;99(22):1685–92. 23894089 10.1136/heartjnl-2013-304163

[16] Desai MS, Mathur B, Eblimit Z, Vasquez H, Taegtmeyer H, Karpen SJ, et al. Bile acid excess induces cardiomyopathy and metabolic dysfunctions in the heart. Hepatology. 2017;65(1):189–201. 27774647 10.1002/hep.28890 PMC5299964

[17] Mayerhofer CC, Ueland T, Broch K, Vincent RP, Cross GF, Dahl CP, et al. Increased secondary/primary bile acid ratio in chronic heart failure. J Cardiac Fail. 2017;23(9):666–71. 10.1016/j.cardfail.2017.06.007 28688889

[18] Huang Y, Zhou M, Sun H, Wang Y. Branched-chain amino acid metabolism in heart disease: an epiphenomenon or a real culprit? Cardiovasc Res. 2011;90(2):220–3. 21502372 10.1093/cvr/cvr070 PMC3078803

[19] Uddin GM, Zhang L, Shah S, Fukushima A, Wagg CS, Gopal K, et al. Impaired branched chain amino acid oxidation contributes to cardiac insulin resistance in heart failure. Cardiovasc Diabetol. 2019;18(1):1–12. 31277657 10.1186/s12933-019-0892-3 PMC6610921

[20] Li Y, Xiong Z, Yan W, Gao E, Cheng H, Wu G, et al. Branched chain amino acids exacerbate myocardial ischemia/reperfusion vulnerability via enhancing GCN2/ATF6/PPAR-α pathway-dependent fatty acid oxidation. Theranostics. 2020;10(12):5623. 32373236 10.7150/thno.44836 PMC7196282

[21] Walejko JM, Christopher BA, Crown SB, Zhang GF, Pickar-Oliver A, Yoneshiro T, et al. Branched-chain α-ketoacids are preferentially reaminated and activate protein synthesis in the heart. Nat Commun. 2021;12(1):1–14. 33723250 10.1038/s41467-021-21962-2 PMC7960706

[22] Tobias DK, Lawler PR, Harada PH, Demler OV, Ridker PM, Manson JE, et al. Circulating branched-chain amino acids and incident cardiovascular disease in a prospective cohort of US women. Circulation. 2018;11(4):e002157. 29572205 10.1161/CIRCGEN.118.002157 PMC5880282

[23] Bastin M, Andreelli F. The gut microbiota and diabetic cardiomyopathy in humans. Diabetes Metab. 2020;46(3):197–202. 31678397 10.1016/j.diabet.2019.10.003

[24] Agus A, Clément K, Sokol H. Gut microbiota-derived metabolites as central regulators in metabolic disorders. Gut. 2021;70(6):1174–82. 33272977 10.1136/gutjnl-2020-323071 PMC8108286

[25] Fan Y, Pedersen O. Gut microbiota in human metabolic health and disease. Nat Rev Microbiol. 2021;19(1):55–71. 32887946 10.1038/s41579-020-0433-9

[26] Han H, Yi B, Zhong R, Wang M, Zhang S, Ma J, et al. From gut microbiota to host appetite: gut microbiota-derived metabolites as key regulators. Microbiome. 2021;9(1):1–16. 34284827 10.1186/s40168-021-01093-y PMC8293578

[27] Lee Y, Wiriyasermkul P, Jin C, Quan L, Ohgaki R, Okuda S, et al. Cryo-EM structure of the human L-type amino acid transporter 1 in complex with glycoprotein CD98hc. Nat Struct Mol Biol. 2019;26(6):510–7. 31160781 10.1038/s41594-019-0237-7

[28] Neinast MD, Jang C, Hui S, Murashige DS, Chu Q, Morscher RJ, et al. Quantitative analysis of the whole-body metabolic fate of branched-chain amino acids. Cell Metab. 2019;29(2):417–29. 30449684 10.1016/j.cmet.2018.10.013 PMC6365191

[29] Plitzko B, Loesgen S. Measurement of oxygen consumption rate (OCR) and extracellular acidification rate (ECAR) in culture cells for assessment of the energy metabolism. Bio-Protoc. 2018;8(10):e2850–e2850. 34285967 10.21769/BioProtoc.2850 PMC8275291

[30] Chiba A, Watanabe-Takano H, Miyazaki T, Mochizuki N. Cardiomyokines from the heart. Cell Mol Life Sci. 2018;75(8):1349–62. 29238844 10.1007/s00018-017-2723-6 PMC11105766

[31] Badman MK, Pissios P, Kennedy AR, Koukos G, Flier JS, Maratos-Flier E. Hepatic fibroblast growth factor 21 is regulated by PPARα and is a key mediator of hepatic lipid metabolism in ketotic states. Cell Metab. 2007;5(6):426–37. 17550778 10.1016/j.cmet.2007.05.002

[32] Oishi K, Uchida D, Ishida N. Circadian expression of FGF21 is induced by PPARα activation in the mouse liver. FEBS Lett. 2008;582:3639–42. 18840432 10.1016/j.febslet.2008.09.046

[33] Cummings NE, Williams EM, Kasza I, Konon EN, Schaid MD, Schmidt BA, et al. Restoration of metabolic health by decreased consumption of branched-chain amino acids. J Physiol. 2018;596(4):623–45. 29266268 10.1113/JP275075 PMC5813603

[34] Yu D, Richardson NE, Green CL, Spicer AB, Murphy ME, Flores V, et al. The adverse metabolic effects of branched-chain amino acids are mediated by isoleucine and valine. Cell Metab. 2021;33(5):905–22. 33887198 10.1016/j.cmet.2021.03.025 PMC8102360

[35] Yan R, Zhao X, Lei J, Zhou Q. Structure of the human LAT1-4F2hc heteromeric amino acid transporter complex. Nature. 2019;568(7750):127–30. 30867591 10.1038/s41586-019-1011-z

[36] Anderson EJ, Katunga LA, Willis MS. Mitochondria as a source and target of lipid peroxidation products in healthy and diseased heart. Clin Exp Pharmacol Physiol. 2012;39(2):179–93. 22066679 10.1111/j.1440-1681.2011.05641.x PMC3827773

[37] Fisher FM, Maratos-Flier E. Understanding the physiology of FGF21. Annu Rev Physiol. 2016;78(1):223–41. 26654352 10.1146/annurev-physiol-021115-105339

[38] Sands MS. AAV-mediated liver-directed gene therapy. Methods Mol Biol. 2011;807:141–57. 22034029 10.1007/978-1-61779-370-7_6 PMC4118577

[39] Fornes O, Castro-Mondragon JA, Khan A, Van der Lee R, Zhang X, Richmond PA, et al. JASPAR 2020: update of the open-access database of transcription factor binding profiles. Nucleic Acids Res. 2020;48:D87–92. 31701148 10.1093/nar/gkz1001 PMC7145627

[40] Jeon BN, Yoon JH, Kim MK, Choi WI, Koh DI, Hur B, et al. Zbtb7c is a molecular ‘off’ and ‘on’ switch of Mmp gene transcription. BBA-Gene Regulatory Mechanisms. 2016;1859(11):1429–39. 27646874 10.1016/j.bbagrm.2016.09.004

[41] Choi WI, Yoon JH, Choi SH, Jeon BN, Kim H, Hur MW. Proto-oncoprotein Zbtb7c and SIRT1 repression: implications in high-fat diet-induced and age-dependent obesity. Exp Mol Med. 2021;53(5):917–32. 34017061 10.1038/s12276-021-00628-5 PMC8178412

[42] Choi WI, Yoon JH, Song JY, Jeon BN, Park JM, Koh DI, et al. Zbtb7c is a critical gluconeogenic transcription factor that induces glucose-6-phosphatase and phosphoenylpyruvatecarboxykinase 1 genes expression during mice fasting. BBA-Gene Regulatory Mechanisms. 2019;1862(6):643–56. 30959128 10.1016/j.bbagrm.2019.04.001

[43] Neinast M, Murashige D, Arany Z. Branched chain amino acids. Annu Rev Physiol. 2019;81:139–64. 30485760 10.1146/annurev-physiol-020518-114455 PMC6536377

[44] Son SM, Park SJ, Stamatakou E, Vicinanza M, Menzies FM, Rubinsztein DC. Leucine regulates autophagy via acetylation of the mTORC1 component raptor. Nat Commun. 2020;11(1):1–13. 32561715 10.1038/s41467-020-16886-2 PMC7305105

[45] Lynch CJ, Adams SH. Branched-chain amino acids in metabolic signalling and insulin resistance. Nat Rev Endocrinol. 2014;10(12):723–36. 25287287 10.1038/nrendo.2014.171 PMC4424797

[46] Bai J, Greene E, Li W, Kidd MT, Dridi S. Branched-chain amino acids modulate the expression of hepatic fatty acid metabolism-related genes in female broiler chickens. Mol Nutr Food Res. 2015;59(6):1171–81. 25787688 10.1002/mnfr.201400918

[47] Zhenyukh O, Civantos E, Ruiz-Ortega M, Sánchez MS, Vazquez C, Peiro C, et al. High concentration of branched-chain amino acids promotes oxidative stress, inflammation and migration of human peripheral blood mononuclear cells via mTORC1 activation. Free Radical Biol Med. 2017;104:165–77. 28089725 10.1016/j.freeradbiomed.2017.01.009

[48] D’Antona G, Ragni M, Cardile A, Tedesco L, Dossena M, Bruttini F, et al. Branched-chain amino acid supplementation promotes survival and supports cardiac and skeletal muscle mitochondrial biogenesis in middle-aged mice. Cell Metab. 2010;12(4):362–72. 20889128 10.1016/j.cmet.2010.08.016

[49] Bloomgarden Z. Diabetes and branched-chain amino acids: What is the link? J Diabetes. 2018;10(5):350–2. 29369529 10.1111/1753-0407.12645

[50] Tysoe O. BCAA maintain white adipose tissue. Nat Rev Endocrinol. 2022;18:194. 35132246 10.1038/s41574-022-00645-y

[51] Peng H, Wang Y, Luo W. Multifaceted role of branched-chain amino acid metabolism in cancer. Oncogene. 2020;39(44):6747–56. 32978521 10.1038/s41388-020-01480-z PMC7606751

[52] Dutta T, Chai HS, Ward LE, Ghosh A, Persson XMT, Ford GC, et al. Concordance of changes in metabolic pathways based on plasma metabolomics and skeletal muscle transcriptomics in type 1 diabetes. Diabetes. 2012;61(5):1004–16. 22415876 10.2337/db11-0874 PMC3331761

[53] Iwasa M, Ishihara T, Mifuji-Moroka R, Fujita N, Kobayashi Y, Hasegawa H, et al. Elevation of branched-chain amino acid levels in diabetes and NAFL and changes with antidiabetic drug treatment. Obes Res Clin Pract. 2015;9(3):293–7. 25649191 10.1016/j.orcp.2015.01.003

[54] Knebel B, Strassburger K, Szendroedi J, Kotzka J, Scheer M, Nowotny B, et al. Specific metabolic profiles and their relationship to insulin resistance in recent-onset type 1 and type 2 diabetes. J Clin Endocrinol Metab. 2016;101(5):2130–40. 26829444 10.1210/jc.2015-4133

[55] Wolak-Dinsmore J, Gruppen EG, Shalaurova I, Matyus SP, Grant RP, Gegen R, et al. A novel NMR-based assay to measure circulating concentrations of branched-chain amino acids: Elevation in subjects with type 2 diabetes mellitus and association with carotid intima media thickness. Clin Biochem. 2018;54:92–9. 29432757 10.1016/j.clinbiochem.2018.02.001

[56] Cheng YW, Alhaffar D, Saha S, Khanna S, Bohm M, Phelps E, et al. Fecal microbiota transplantation is safe and effective in patients with Clostridioides difficile infection and cirrhosis. Clin Gastroenterol Hepatol. 2021;19(8):1627–34. 32645451 10.1016/j.cgh.2020.06.051 PMC8856132

[57] Costello SP, Hughes PA, Waters O, Bryant RV, Vincent AD, Blatchford P, et al. Effect of fecal microbiota transplantation on 8-week remission in patients with ulcerative colitis: a randomized clinical trial. JAMA. 2019;321(2):156–64. 30644982 10.1001/jama.2018.20046 PMC6439766

[58] Sun J, Xu J, Ling Y, Wang F, Gong T, Yang C, et al. Fecal microbiota transplantation alleviated Alzheimer’s disease-like pathogenesis in APP/PS1 transgenic mice. Transl Psychiatry. 2019;9(1):1–13. 31383855 10.1038/s41398-019-0525-3 PMC6683152

[59] Hu XF, Zhang WY, Wen Q, Chen WJ, Wang ZM, Chen J, et al. Fecal microbiota transplantation alleviates myocardial damage in myocarditis by restoring the microbiota composition. Pharmacol Res. 2019;139:412–21. 30508676 10.1016/j.phrs.2018.11.042

[60] Kaźmierczak-Siedlecka K, Daca A, Fic M, van de Wetering T, Folwarski M, Makarewicz W. Therapeutic methods of gut microbiota modification in colorectal cancer management–fecal microbiota transplantation, prebiotics, probiotics, and synbiotics. Gut Microbes. 2020;11(6):1518–30. 32453670 10.1080/19490976.2020.1764309 PMC7524363

[61] Yu EW, Gao L, Stastka P, Cheney MC, Mahabamunuge J, Torres Soto M, et al. Fecal microbiota transplantation for the improvement of metabolism in obesity: the FMT-TRIM double-blind placebo-controlled pilot trial. PLoS Med. 2020;17(3):e1003051. 32150549 10.1371/journal.pmed.1003051 PMC7062239

[62] De Groot P, Nikolic T, Pellegrini S, Sordi V, Imangaliyev S, Rampanelli E, et al. Faecal microbiota transplantation halts progression of human new-onset type 1 diabetes in a randomised controlled trial. Gut. 2021;70(1):92–105. 33106354 10.1136/gutjnl-2020-322630 PMC7788262

[63] Martin A, Ecklu-Mensah G, Ha CW, Hendrick G, Layman DK, Gilbert J, Devkota S. Gut microbiota mediate the FGF21 adaptive stress response to chronic dietary protein-restriction in mice. Nat Commun. 2021;12(1):1–11. 34158480 10.1038/s41467-021-24074-z PMC8219803

[64] Kundu P, Lee HU, Garcia-Perez I, Tay EXY, Kim H, Faylon LE, et al. Neurogenesis and prolongevity signaling in young germ-free mice transplanted with the gut microbiota of old mice. Sci Transl Med. 2019;11(518):eaau4760. 31723038 10.1126/scitranslmed.aau4760

[65] Planavila A, Redondo I, Hondares E, Vinciguerra M, Munts C, Iglesias R, et al. Fibroblast growth factor 21 protects against cardiac hypertrophy in mice. Nat Commun. 2013;4(1):1–12. 10.1038/ncomms3019 23771152

[66] Planavila A, Redondo-Angulo I, Ribas F, Garrabou G, Casademont J, Giralt M, Villarroya F. Fibroblast growth factor 21 protects the heart from oxidative stress. Cardiovasc Res. 2015;106(1):19–31. 25538153 10.1093/cvr/cvu263

[67] Zhang C, Huang Z, Gu J, Yan X, Lu X, Zhou S, et al. Fibroblast growth factor 21 protects the heart from apoptosis in a diabetic mouse model via extracellular signal-regulated kinase 1/2-dependent signalling pathway. Diabetologia. 2015;58(8):1937–48. 26040473 10.1007/s00125-015-3630-8

[68] Yang H, Feng A, Lin S, Yu L, Lin X, Yan X, et al. Fibroblast growth factor-21 prevents diabetic cardiomyopathy via AMPK-mediated antioxidation and lipid-lowering effects in the heart. Cell Death Dis. 2018;9(2):1–14. 29445083 10.1038/s41419-018-0307-5 PMC5833682

[69] Hur MW, Yoon JH, Kim MY, Ko H, Jeon BN. Kr-POK (ZBTB7c) regulates cancer cell proliferation through glutamine metabolism. BBA-Gene Regulatory Mechanisms. 2017;1860(8):829–38. 28571744 10.1016/j.bbagrm.2017.05.005

[70] Furman BL. Streptozotocin-induced diabetic models in mice and rats. Curr Protoc Pharmacol. 2015;70(1):5–47. 10.1002/0471141755.ph0547s70 26331889

[71] Zheng H, Ni Z, Cai A, Zhang X, Chen J, Shu Q, Gao H. Balancing metabolome coverage and reproducibility for untargeted NMR-based metabolic profiling in tissue samples through mixture design methods. Anal Bioanal Chem. 2018;410(29):7783–92. 30298192 10.1007/s00216-018-1396-9

[72] Zhang X, Lin Q, Chen J, Wei T, Li C, Zhao L, et al. High glucose-induced cardiomyocyte death may be linked to unbalanced branched-chain amino acids and energy metabolism. Molecules. 2018;23(4):807. 29614759 10.3390/molecules23040807 PMC6017930

[73] Wishart DS, Feunang YD, Marcu A, Guo AC, Liang K, Vázquez-Fresno R, et al. HMDB 4.0: the human metabolome database for 2018. Nucleic Acids Res. 2018;46:D608–17. 29140435 10.1093/nar/gkx1089 PMC5753273

[74] Jin M, Chen Y, Zhao Y, Che L, Ma Y, Li J, Wang Y, Tao H, Ma J, Pan B, et al. Sortase A-aided Escherichia coli expression system for functional osteoprotegerin cysteine-rich domain. Appl Microbiol Biotechnol. 2017;101:4923–33. 28303296 10.1007/s00253-017-8188-6

