# Supplementary material for: BCAA mediated microbiota-liver-heart crosstalk regulates diabetic cardiomyopathy via FGF21
Source: Microbiome. 2024 Aug 24;12:157. doi: 10.1186/s40168-024-01872-3 (PMC11344321; doi:10.1186/s40168-024-01872-3)
Supplement: Supplementary file 2 — Supplementary Material 1: Table S1. Metabolic differences in heart tissue between normal control (Ctrl) and type 1 diabetic (T1D) mice. Table S2. Intracellular metabolic differences of H9C2 cells cultured under normal glucose (NG) and high glucose (HG) conditions. Table S3. Extracellular metabolic differences of H9C2 cells cultured under normal glucose (NG) and high glucose (HG) conditions. Table S4. Amino acid compositions of H9c2 cell culture media. Table S5. Primer pairs used in this study. Table S6. Primer pairs used for ChIP assay in this study. Figure S1. Diabetic cardiomyopathy occurs in streptozotocin (STZ)-induced type 1 diabetic mice. Figure S2. High glucose induces myocardial apoptosis. Figure S3. NMR-based metabolomics profiling. Figure S4. Faecal microbiota transplantation (FMT) partly corrects the gut microbiota in T1D mice. Figure S5. Faecal microbiota transplantation (FMT) reshapes the microbial function in T1D mice. Figure S6. Changes in BCAA catabolism enzymes both in vivo and in vitro. Figure S7. Changes in BCAA and their transporter LAT1 both in vivo and in vitro. Figure S8. Rapamycin alleviates mitochondrial damage and apoptosis of myocardial cells. Figure S9. Faecal microbiota transplantation (FMT) alleviates cardiac injure in T1D mice by reducing LAT1-driven increase in BCAAs and inhibiting mTOR pathway. Figure S10. Excess BCAA decreases FGF21 production both in vivo and in vitro. Figure S11. FGF21 alleviates mitochondrial damage and apoptosis of myocardial cells. Figure S12. FGF21 reduces BCAA in H9c2 cells under high glucose condition. Figure S13. The effect of AAV-mediated FGF21 knockdown on hepatic FGF21 production and cardiac BCAA levels in T1D mice with FMT. [file 40168_2024_1872_MOESM1_ESM.docx]

BCAA mediated microbiota-liver-heart crosstalk regulatesdiabetic cardiomyopathy via FGF21

Hong Zheng^1,†^, Xi Zhang^1,†^, Chen Li^1,†^, Die Wang^1^, Yuying Shen^1^, Jiahui Lu^1^, Liangcai Zhao^1^, Xiaokun Li^1,2^ & Hongchang Gao^1,2,*^

^1^School of Pharmaceutical Sciences, Wenzhou Medical University, Wenzhou 325035, China

^2^Oujiang Laboratory; Institute of Aging, Key Laboratory of Alzheimer's Disease of Zhejiang Province, Wenzhou Medical University, Wenzhou 325035, China

^†^These authors contributed equally.

^*^Correspondence: [gaohc27@wmu.edu.cn](mailto:gaohc27@wmu.edu.cn) (H.C.G.).

**Table S1.** Metabolic differences in heart tissue between normal control (Ctrl) and type 1 diabetic (T1D) mice.

| Metabolite | Ctrl | T1D | P value |
| --- | --- | --- | --- |
| acetate | 2.88±0.48^a^ | 2.82±0.51 | 0.788 |
| alanine | 17.11±1.42 | 18.53±0.62 | 0.030 |
| aspartate | 55.44±21.14 | 65.12±9.39 | 0.331 |
| ADP^b^ | 5.56±0.81 | 2.94±1.31 | <0.001 |
| AMP^c^ | 4.12±0.50 | 1.69±0.71 | <0.001 |
| ATP^d^ | 0.69±0.38 | 0.18±0.08 | 0.003 |
| choline | 13.23±1.21 | 13.72±2.67 | 0.713 |
| creatine | 41.26±0.68 | 34.87±5.37 | 0.006 |
| fumarate | 0.81±0.17 | 0.89±0.14 | 0.348 |
| glutamate | 58.56±1.61 | 62.60±1.92 | 0.002 |
| glutamine | 57.16±1.71 | 60.92±1.99 | 0.002 |
| glycine | 7.60±1.40 | 7.70±1.25 | 0.941 |
| isoleucine | 2.45±0.16 | 3.45±0.85 | 0.004 |
| inosine | 2.80±0.30 | 3.34±0.76 | 0.076 |
| lactate | 131.43±12.49 | 102.65±15.07 | 0.002 |
| leucine | 4.01±0.57 | 6.10±1.51 | 0.002 |
| niacinamide | 0.70±0.04 | 0.63±0.10 | 0.094 |
| taurine | 112.32±4.89 | 121.43±8.58 | 0.021 |
| valine | 1.60±0.16 | 3.02±0.89 | <0.001 |

^a^ data were presented as mean±SD; ^b^ adenosine diphosphate; ^c^ adenosine monophosphate; ^d^ adenosine triphosphate.

**Table S2.** Intracellular metabolic differences of H9C2 cells cultured under normal glucose (NG) and high glucose (HG) conditions.

| Metabolite | NG | HG | P value |
| --- | --- | --- | --- |
| acetate | 5.36±0.38^a^ | 5.08 ±0.15 | 0.392 |
| alanine | 15.81±0.76 | 19.49±1.83 | 0.013 |
| arginine | 28.24±1.41 | 28.58±2.39 | 0.945 |
| aspartate | 5.75±0.60 | 5.16±0.33 | 0.108 |
| ADP^b^ | 4.34±0.39 | 4.09±0.33 | 0.342 |
| AMP^c^ | 0.19±0.03 | 0.17±0.06 | 0.493 |
| ATP^d^ | 1.31±0.08 | 0.97±0.09 | <0.001 |
| betanine | 3.94±0.19 | 6.06±1.85 | 0.052 |
| choline | 5.95±0.45 | 4.57±1.08 | 0.053 |
| creatine | 13.97±1.58 | 12.36±0.77 | 0.137 |
| formate | 0.36±0.09 | 0.53±0.24 | 0.304 |
| fumarate | 0.10±0.03 | 0.10±0.02 | 0.813 |
| glutamate | 65.42±4.46 | 62.06±3.72 | 0.181 |
| glutamine | 26.21±4.18 | 33.67±3.02 | 0.012 |
| glutathione | 19.28±1.28 | 18.73±1.33 | 0.439 |
| glycine | 13.74±1.93 | 17.88±1.73 | 0.333 |
| isoleucine | 6.48±0.20 | 7.86±0.31 | <0.001 |
| lactate | 53.05±10.06 | 70.42±6.68 | 0.008 |
| leucine | 17.07±0.43 | 19.95±0.61 | <0.001 |
| myo-Inositol | 4.21±0.08 | 4.37±0.19 | 0.241 |
| NAD+^e^ | 0.62±0.07 | 0.68±0.03 | 0.202 |
| o-phosphocholine | 32.34±2.81 | 27.88±1.74 | 0.015 |
| p-creatine | 4.43±0.52 | 4.16±0.40 | 0.32 |
| phenylalanine | 2.41±0.20 | 3.13±0.27 | 0.002 |
| sarcosine | 1.08±0.06 | 0.91±0.11 | 0.011 |
| succinate | 2.31±0.21 | 2.27±0.31 | 0.871 |
| tryptophan | 0.36±0.02 | 0.38±0.04 | 0.509 |
| tyrosine | 2.27±0.21 | 2.94±0.30 | 0.004 |
| valine | 7.88±0.29 | 9.55±0.48 | <0.001 |

^a^ data were presented as mean±SD; ^b^ adenosine diphosphate; ^c^ adenosine monophosphate; ^d^ adenosine triphosphate; ^e^nicotinamide adenine dinucleotide.

**Table S3.** Extracellular metabolic differences of H9C2 cells cultured under normal glucose (NG) and high glucose (HG) conditions.

| Metabolite | NG | HG | P value |
| --- | --- | --- | --- |
| acetate | 35.04±4.64^a^ | 38.29±3.57 | 0.250 |
| alanine | 18.61±0.66 | 17.84±0.58 | 0.086 |
| betanine | 1.39±0.08 | 1.89±0.08 | <0.001 |
| choline | 3.05±0.17 | 2.98±0.19 | 0.574 |
| creatine | 0.81±0.02 | 0.77±0.09 | 0.361 |
| formate | 0.61±0.06 | 0.60±0.08 | 0.812 |
| isoleucine | 17.22±0.58 | 14.98±0.83 | 0.001 |
| leucine | 22.96±0.88 | 20.49±1.01 | 0.003 |
| o-phosphocholine | 1.83±0.07 | 2.15±0.11 | 0.001 |
| p-creatine | 0.83±0.04 | 0.67±0.04 | <0.001 |
| phenylalanine | 7.08±0.18 | 6.55±0.11 | <0.001 |
| tyrosine | 0.54±0.02 | 0.48±0.03 | 0.006 |
| tryptophan | 0.54±0.02 | 0.54±0.02 | 0.860 |
| valine | 18.49±0.52 | 16.56±0.52 | <0.001 |

^a^ data were presented as mean±SD.

**Table S4.** Amino acid compositions of H9c2 cell culture media.

| Amino acid (mM) | DMEM | DMEM  (high leucine) | DMEM  (high isoleucine) | DMEM  (high valine) |
| --- | --- | --- | --- | --- |
| L-Leucine | 0.80 | 10.80 | 0.80 | 0.80 |
| L-Isoleucine | 0.80 | 0.80 | 5.80 | 0.80 |
| L-Valine | 0.80 | 0.80 | 0.80 | 5.80 |
| Glycine | 0.40 | 0.40 | 0.40 | 0.40 |
| L-Alanyl-Glutamine | 3.97 | 3.97 | 3.97 | 3.97 |
| L-Arginine hydrochloride | 0.40 | 0.40 | 0.40 | 0.40 |
| L-Cystine | 0.15 | 0.15 | 0.15 | 0.15 |
| L-Histidine hydrochloride | 0.20 | 0.20 | 0.20 | 0.20 |
| L-Lysine hydrochloride | 0.80 | 0.80 | 0.80 | 0.80 |
| L-Methionine | 0.20 | 0.20 | 0.20 | 0.20 |
| L-Phenylalanine | 0.40 | 0.40 | 0.40 | 0.40 |
| L-Serine | 0.40 | 0.40 | 0.40 | 0.40 |
| L-Threonine | 0.80 | 0.80 | 0.80 | 0.80 |
| L-Tryptophan | 0.08 | 0.08 | 0.08 | 0.08 |
| L-Tyrosine | 0.40 | 0.40 | 0.40 | 0.40 |

**Table S5.** Primer pairs used in this study.

| Gene | Species | Forward | Reverse |
| --- | --- | --- | --- |
| LAT1 | Mouse | GCTTGTCTTGCTTCGGCTCT | CTGTGGGTGGATCATGGAGA |
| BCAT | Mouse | CCCATCGTACCTCTTTCACCC | GGGAGCGTGGGAATACGTG |
| BCKD | Mouse | CTCCTGTTGGGACGATCTGG | CATTGGGCTGGATGAACTCAA |
| BCKDK | Mouse | ACATCAGCCACCGATACACAC | GAGGCGAACTGAGGGCTTC |
| PP2Cm | Mouse | ATGTTATCAGCGGCCTTCATTAC | GTGGAGAAGTAGCAGGCAGG |
| FGF21 | Mouse | GTGTCAAAGCCTCTAGGTTTCTT | GGTACACATTGTAACCGTCCTC |
| PPARα | Mouse | GTGTACGACAAGTGTGATCGG | TTGGCATTCTTCCAAAGCGAA |
| PPARγ | Mouse | TGGCATCTCTGTGTCAACCATG | GCATGGTGCCTTCGCTGA |
| Ehhadh | Mouse | ATACAGCGATACCAGAAGCCA | GTAGAAGCTGCGTTCCTCTTG |
| Fabp3 | Mouse | CTCATCCATGTGCAGAAGTGG | GCCTCCTTCTCATAAGTCCGA |
| ZBTB7C | Mouse | CCCATCTGCCACAAGGTCAT | TGCTTCCGCATGTGGATCTT |
| GAPDH | Mouse | AGGTCGGTGTGAACGGATTTG | TGTAGACCATGTAGTTGAGGTCA |
| LAT1 | Rat | CCAGATGGGAAAGGACATAGGA | TCCCCACGTCCAGATTGGT |
| BCAT | Rat | TCCAGAACCTCACACTGCAC | CAGAGGCGCCTTGCAGAA |
| BCKD | Rat | CTCGGGGCTTGGCTAGATTC | GAACTGGGGCTTGTCATCCA |
| BCKDK | Rat | TTGGCATCATCTGCACTCGT | GCCCATTGATGCGGACTCTA |
| PP2Cm | Rat | GAAACTGTCCTGACCTTGGC | CTGTTGCAGTAGTCCCAGAGG |
| ZBTB7C | Rat | CCCATCTGCCACAAGGTCAT | TGCTTCCGCATGTGGATCTT |
| GAPDH | Rat | GGCATCGTGGAAGGGCTCATGAC | ATGCCAGTGAGCTTCCCGTTCAGC |

**Table S6.** Primer pairs used for ChIP assay in this study.

| Gene | Species | Forward | Reverse |
| --- | --- | --- | --- |
| NC | Rat | GAGGAGGGACACCTACAGAGAC | GCACCTTGGAGCAGTGACATC |
| LAT1 | Rat | CTGTGGGGTGAAGCACGTTA | CCCTGACCTATTCTTCCCTGC |


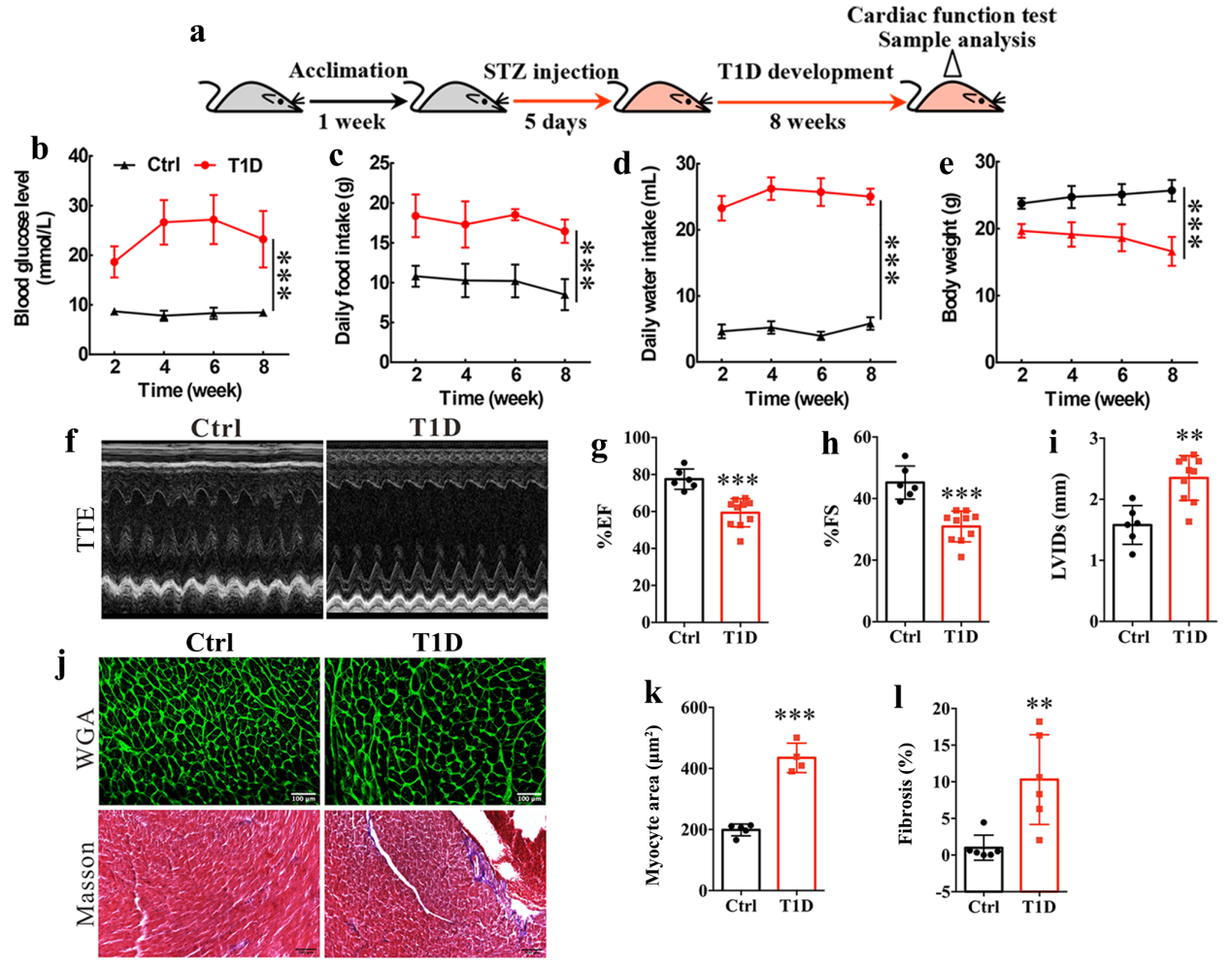


**Figure S1.** Diabetic cardiomyopathy occurs in streptozotocin (STZ)-induced type 1 diabetic mice.(a) Flow diagram of experiment: After 1 week of acclimation, mice were injected with streptozocin(STZ) for 5 days to induce type 1 diabetic (T1D) mice, and then subjected to cardiac function test with echocardiography and sample analysis after 8 weeks (n=6-10 mice per group). The levels of (b) blood glucose, (c) daily food intake, (d) daily water intake and (e) body weight in control (Ctrl) and T1D mice. (f) Representative M-mode echocardiographs in Ctrl and T1D mice. (g) Left ventricular ejection fraction (%EF), (h) left ventricular fractional shortening (%FS) and (i) left ventricular internal dimension at systole (LVIDs) in Ctrl and T1D mice. (j) Representative histological images of wheat germ agglutinin (WGA) and Masson staining (bar=100 μm) and the corresponding quantitative results to indicate (k) cardiomyocyte size and (l) degree of fibrosis in Ctrl and T1D mice. The difference between two groups with time was analyzed with a repeated measure ANOVA. The difference between two groups was analyzed by using two-tailed unpaired student’s t test with Bonferroni correction. Significant level: **p<0.01; ***p<0.001.


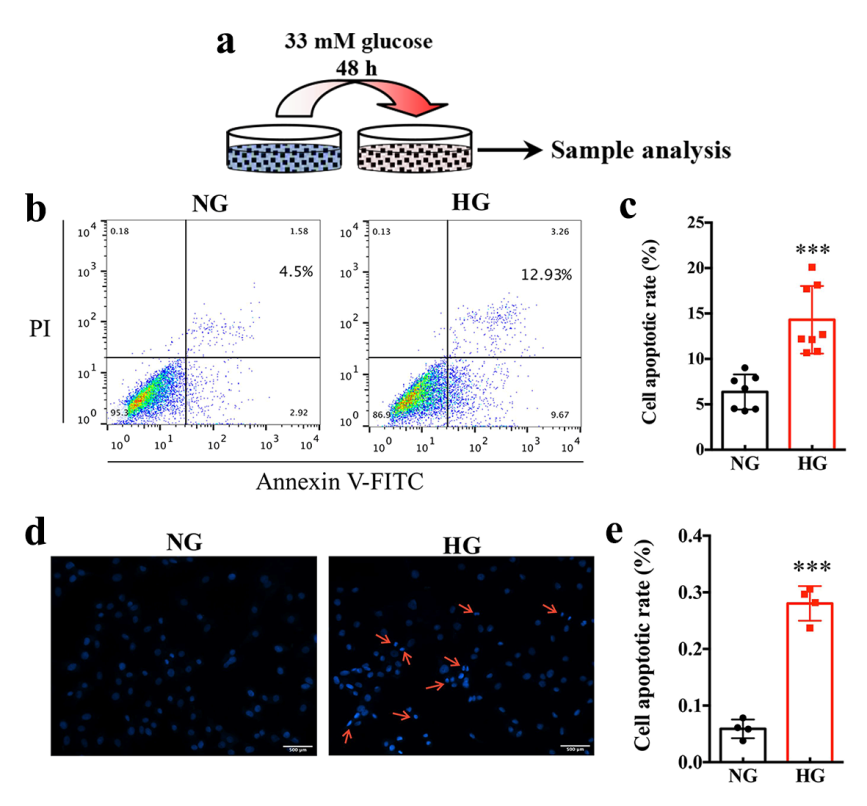


**Figure S2.** High glucose induces myocardial apoptosis. (a) H9c2 cells were cultured under a high glucose condition (33 mM glucose) for 48 h and then collected for sample analysis. (b) Flow cytometry analysis in H9c2 cells under normal glucose (NG) and high glucose (HG) conditions and (c) the corresponding quantitative data. (d) Tunel staining in H9c2 cells under NG and HG conditions and (c) the corresponding quantitative data. The difference between two groups was analyzed by using two-tailed unpaired student’s t test with Bonferroni correction. Significant level: ***p<0.001.


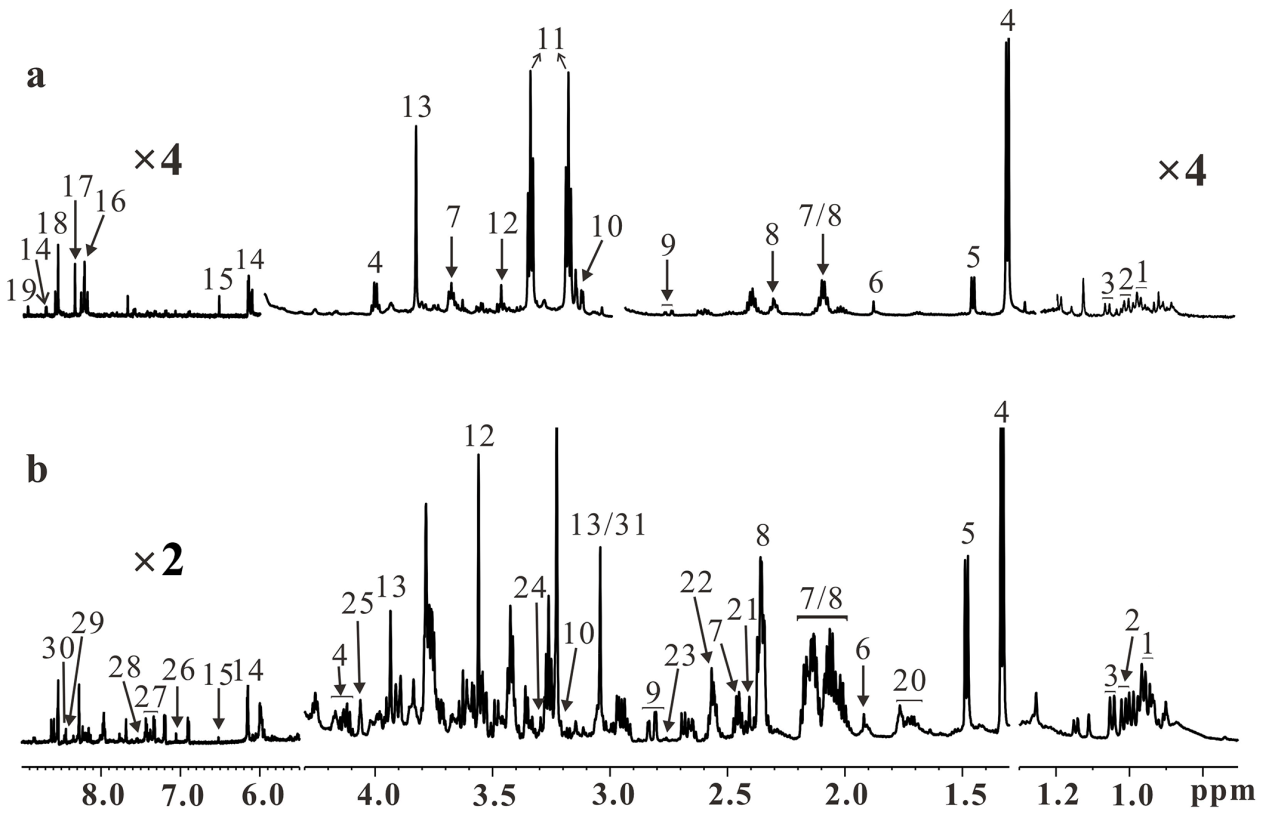


**Figure S3.** NMR-based metabolomics profiling. Typical ^1^H NMR spectra obtained from (a) the heart tissue of control mice and (b) H9c2 cells. Metabolite number: 1, leucine; 2, isoleucine; 3, valine; 4. lactate; 5, alanine; 6, acetate; 7, glutamine; 8, glutamate; 9, aspartate; 10, choline; 11, taurine; 12, glycine; 13, creatine; 14, ATP; 15, fumarate; 16, AMP; 17, inosine; 18, ADP; 19, niacinamide; 20, arginine; 21, succinate; 22, glutathione; 23, sarcosine; 24, betaine; 25, myo-inositol; 26, tyrosine; 27, phenylalanine; 28, tryptophan; 29, NAD+; 30, formate; 31, phosphocreatine. ×2, magnification 2 times; ×4, magnification 4 times.


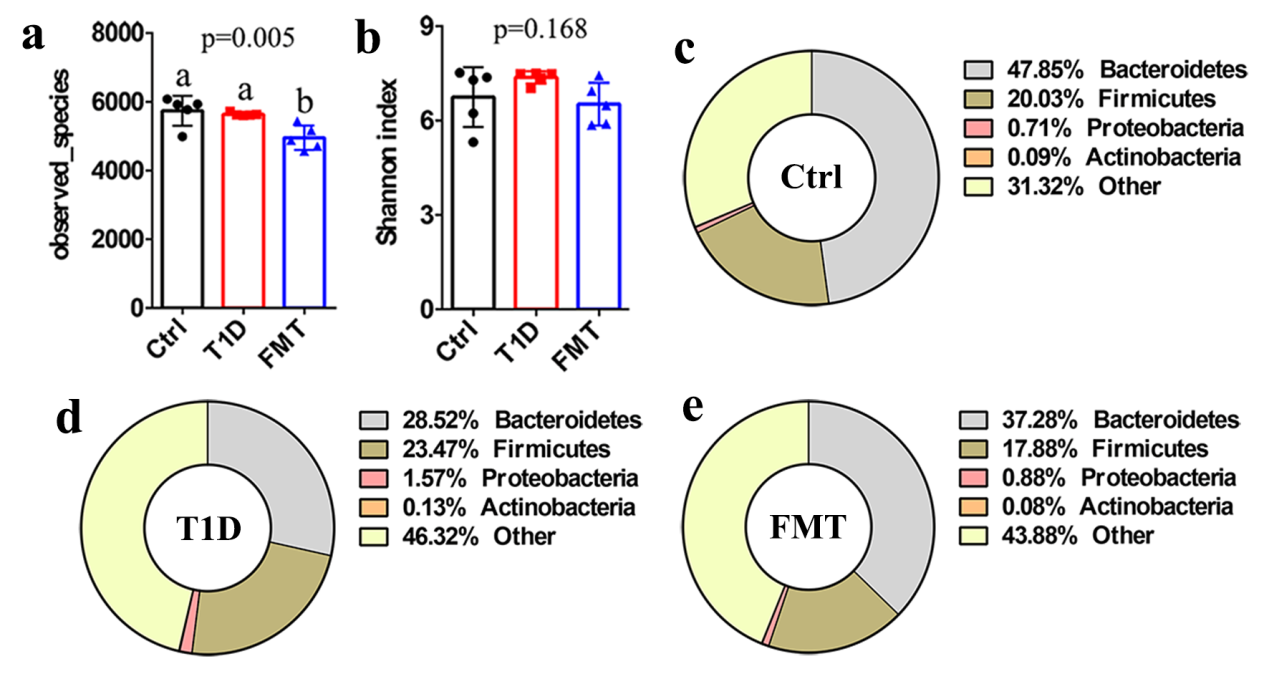


**Figure S4.** Faecal microbiota transplantation (FMT) partly corrects the gut microbiota in T1D mice. (a) The observed species and (b) Shannon index in control (Ctrl), type 1 diabetic (T1D) mice and T1D mice with FMT. The percentage of the gut microbiota at the phylum level of (c) Ctrl, (d) T1D and (e) FMT mice. The differences among three groups were analyzed by using one-way ANOVA with Bonferroni’s multiple comparisons test, and different lowercase codes represent a statistically significant difference (p<0.05).


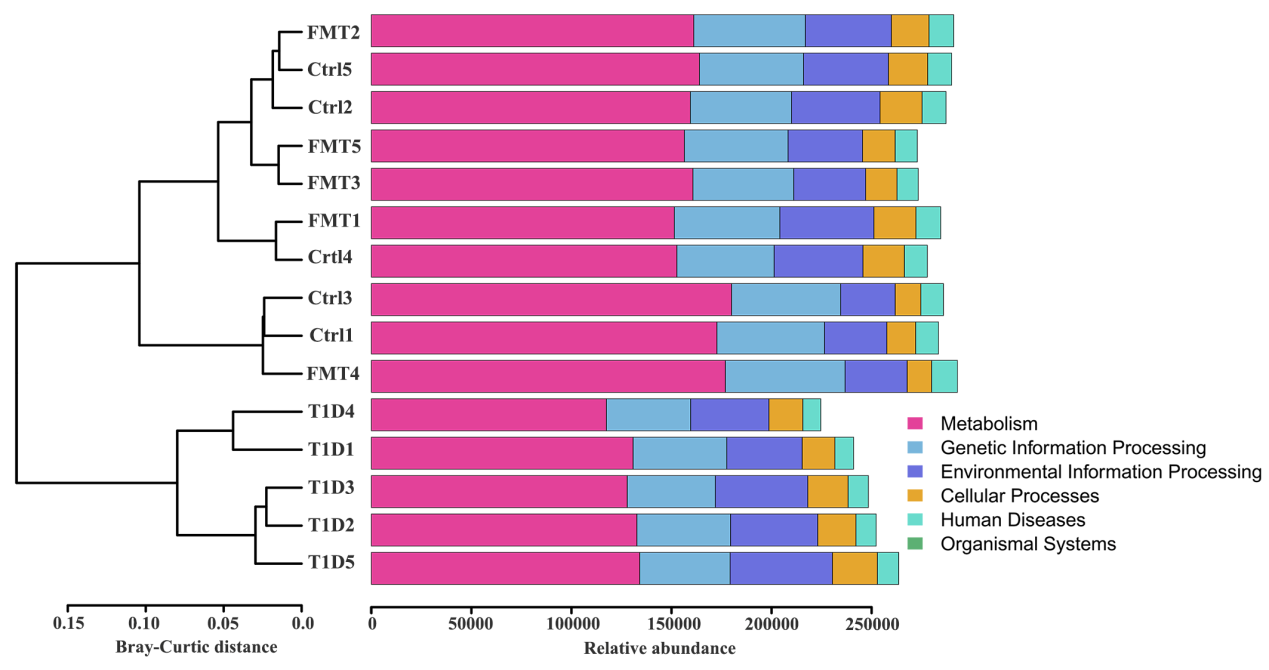


**Figure S5.** Faecal microbiota transplantation (FMT) reshapes the microbial function in T1D mice. Metagenomic reads were annotated according to the KEGG database and cluster analysis based on Bray-Curtic distance was performed to examine the difference in the microbial function among control (Ctrl), type 1 diabetic (T1D) mice and T1D mice with FMT.


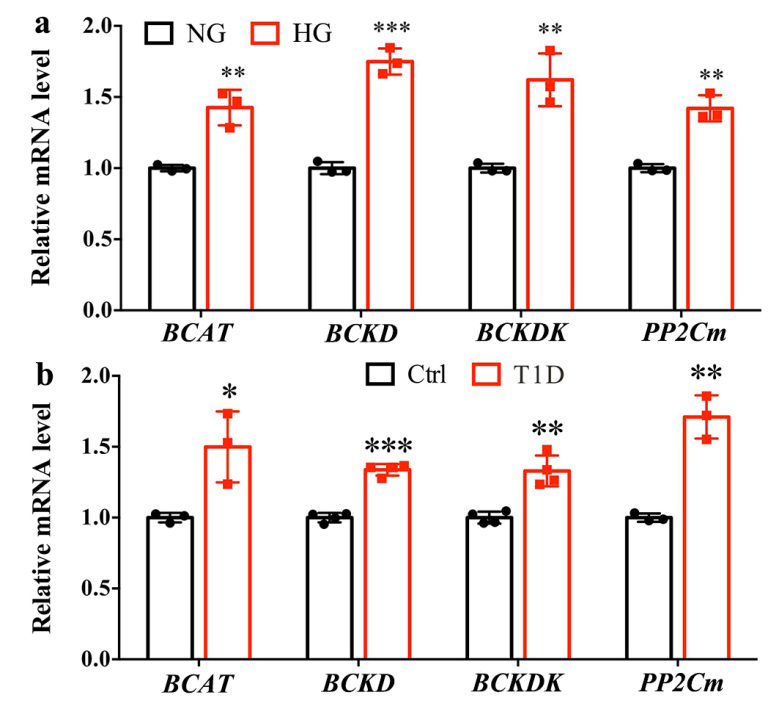


**Figure S6.** Changes in BCAA catabolism enzymes both in vivo and in vitro. (a) The levels of BCAT, BCKD, BCKDK and PP2Cm in H9c2 cells under normal glucose (NG) and high glucose (HG) conditions. (b) The levels of BCAT, BCKD, BCKDK and PP2Cm in control (Ctrl) and type 1 diabetic (T1D) mice. The difference between two groups was analyzed by using two-tailed unpaired student’s t test with Bonferroni correction. Significant level: *p<0.05; **p<0.01; ***p<0.001.


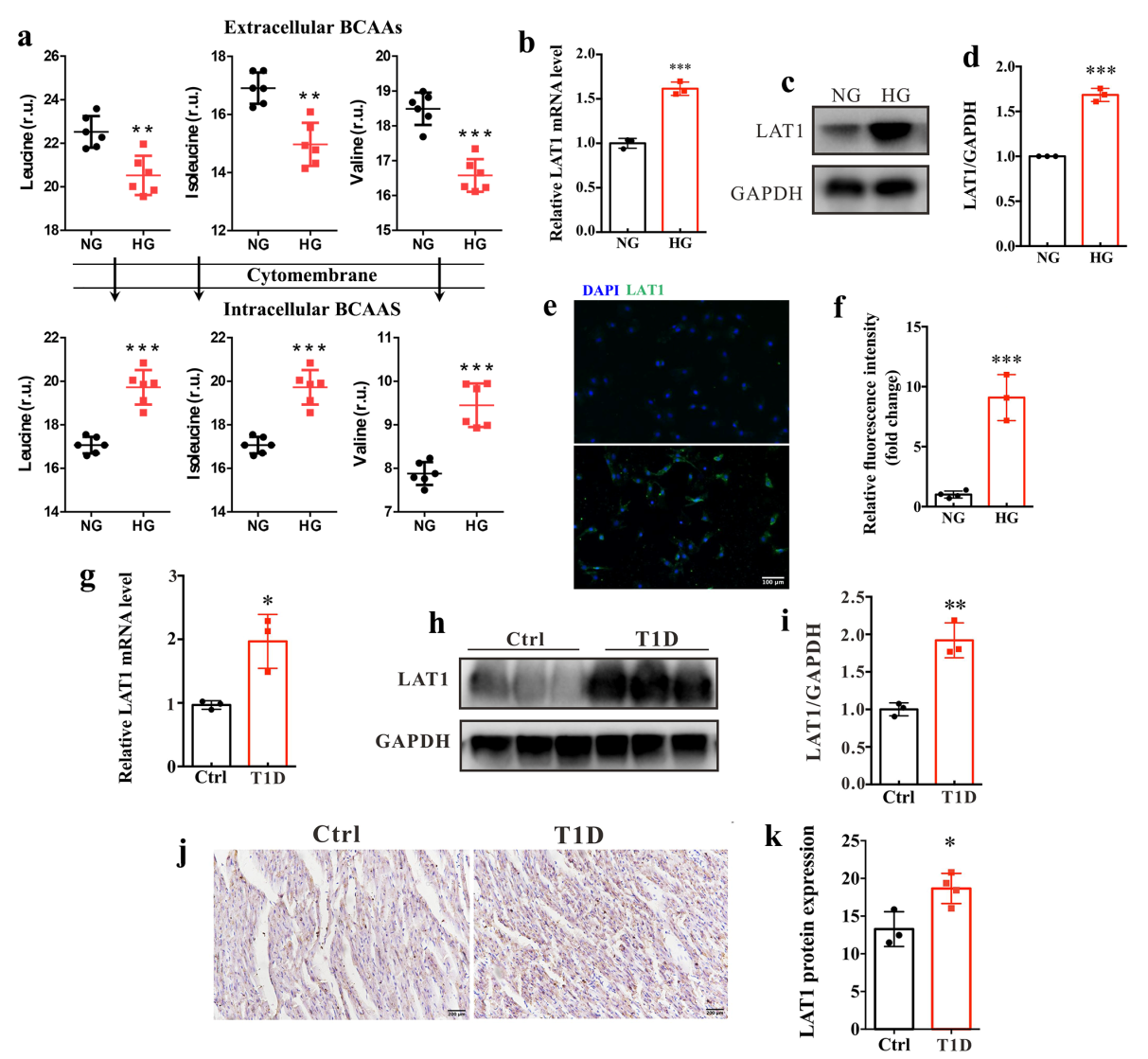


**Figure S7.** Changes in BCAA and their transporter LAT1 both in vivo and in vitro. (a) Intracellular and extracellular BCAA levels in H9c2 cells under normal glucose (NG) and high glucose (HG) conditions.(b) Relative LAT1 mRNA level in H9c2 cells under NG and HG conditions. (c) Western blotting showing the expression levels of LAT1 in H9c2 cells under NG and HG conditions and (d) the corresponding quantitative data. (e) Representative images of LAT1 staining (bar=100 μm) and (f) the corresponding quantitative data to show the level of LAT1 in H9c2 cells under NG and HG conditions. (g) Relative LAT1 mRNA level in the heart of control (Ctrl) and type 1 diabetic (T1D) mice.(h) Western blotting showing the expression levels of LAT1 in the heart of Ctrl and T1D mice and (i) the corresponding quantitative data. (j) Representative histological images of LAT1 staining (bar=100 μm) and (k) the corresponding quantitative data to show the level of LAT1 in the heart of Ctrl and T1D mice. The difference between two groups was analyzed by using two-tailed unpaired student’s t test with Bonferroni correction. Significant level: *p<0.05; **p<0.01; ***p<0.001.


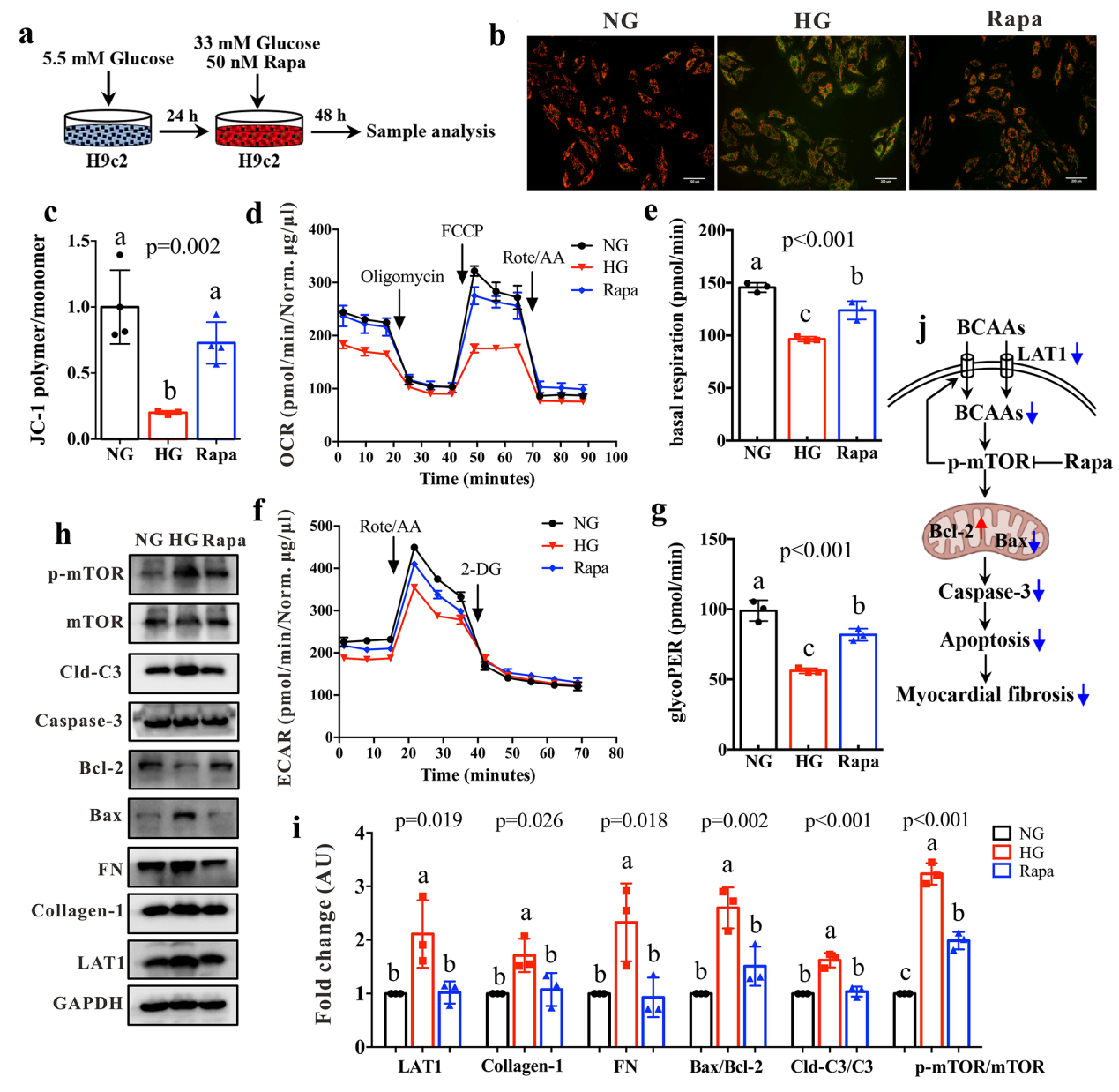


**Figure S8.** Rapamycin alleviates mitochondrial damage and apoptosis of myocardial cells. (a) H9c2 cells were treated with rapamycin (Rapa, 50 nM) and cultured under a high glucose condition (33 mM glucose) for 48 h and then collected for sample analysis. (b) Representative images ofJC-1 staining and (c) the ratio of JC-1 polymer/monomer to indicate the mitochondrial membrane potential in H9c2 cells under normal glucose (NG) and high glucose (HG) conditions as well as Rapa-treated H9c2 cells under HG condition. (d) The curve of oxygen consumption rate (OCR) and (e) the corresponding quantitative data to indicate mitochondrial respiration in H9c2 cells under NG and HG conditions and Rapa-treated H9c2 cells under HG condition. (f) The curve of extracellular acidification rate (ECAR) and (g) the corresponding quantitative data to indicate mitochondrial glycolysis in H9c2 cells under NG and HG conditions and Rapa-treated H9c2 cells under HG condition. (h) Western blotting showing the expression levels of LAT1, Collagen-1, fibronectin (FN), Bax, Bcl-2, caspase-3, cleaved caspase-3 (Cld-C3), mTOR and p-mTOR in H9c2 cells under NG and HG conditions and Rapa-treated H9c2 cells under HG condition and (i) the corresponding quantitative results. (j) The potential role of mTOR in mitochondrial function and myocardial fibrosis in H9c2 cells. The differences among three groups were analyzed by using one-way ANOVA with Bonferroni’s multiple comparisons test, and different lowercase codes represent a statistically significant difference (p<0.05).


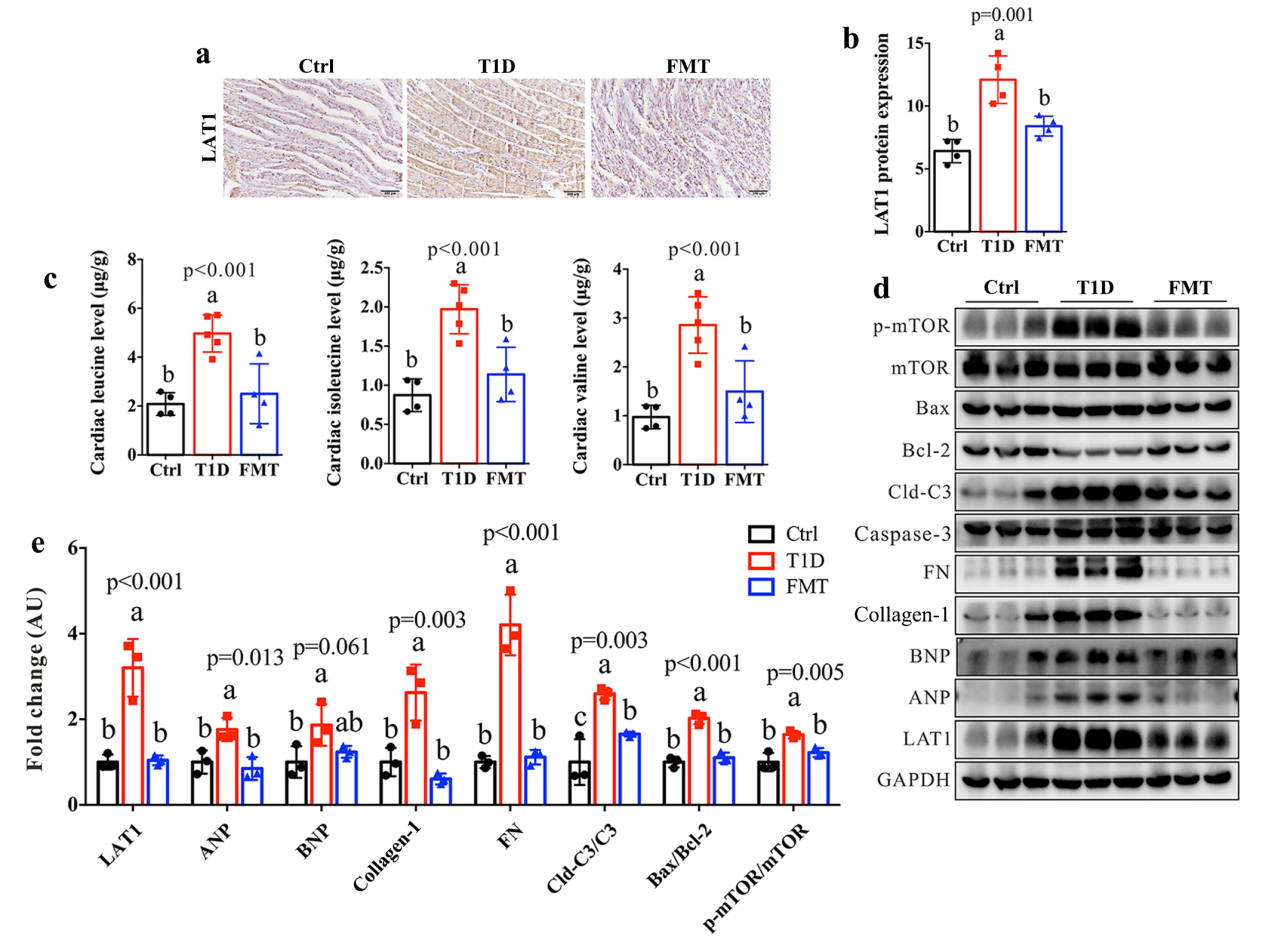


**Figure S9.** Faecal microbiota transplantation (FMT) alleviates cardiac injure in T1D mice by reducing LAT1-driven increase in BCAAs and inhibiting mTOR pathway. (a) Representative histological images of LAT1 staining (bar=100 μm) and (b) the corresponding quantitative data to show the level of LAT1 in the heart of control (Ctrl), type 1 diabetic (T1D) mice and T1D mice with FMT. (c) The levels of leucine, isoleucine and valine in the heart of Ctrl, T1D and FMT mice. (d) Western blotting showing the expression levels of LAT1, atrial natriuretic peptide (ANP), B-type natriuretic peptide (BNP), Collagen-1, fibronectin (FN), Bax, Bcl-2, caspase-3, cleaved caspase-3 (Cld-C3), mTOR and p-mTOR in Ctrl, T1D and FMT mice and (e) the corresponding quantitative data. The differences among three groups were analyzed by using one-way ANOVA with Bonferroni’s multiple comparisons test, and different lowercase codes represent a statistically significant difference (p<0.05).


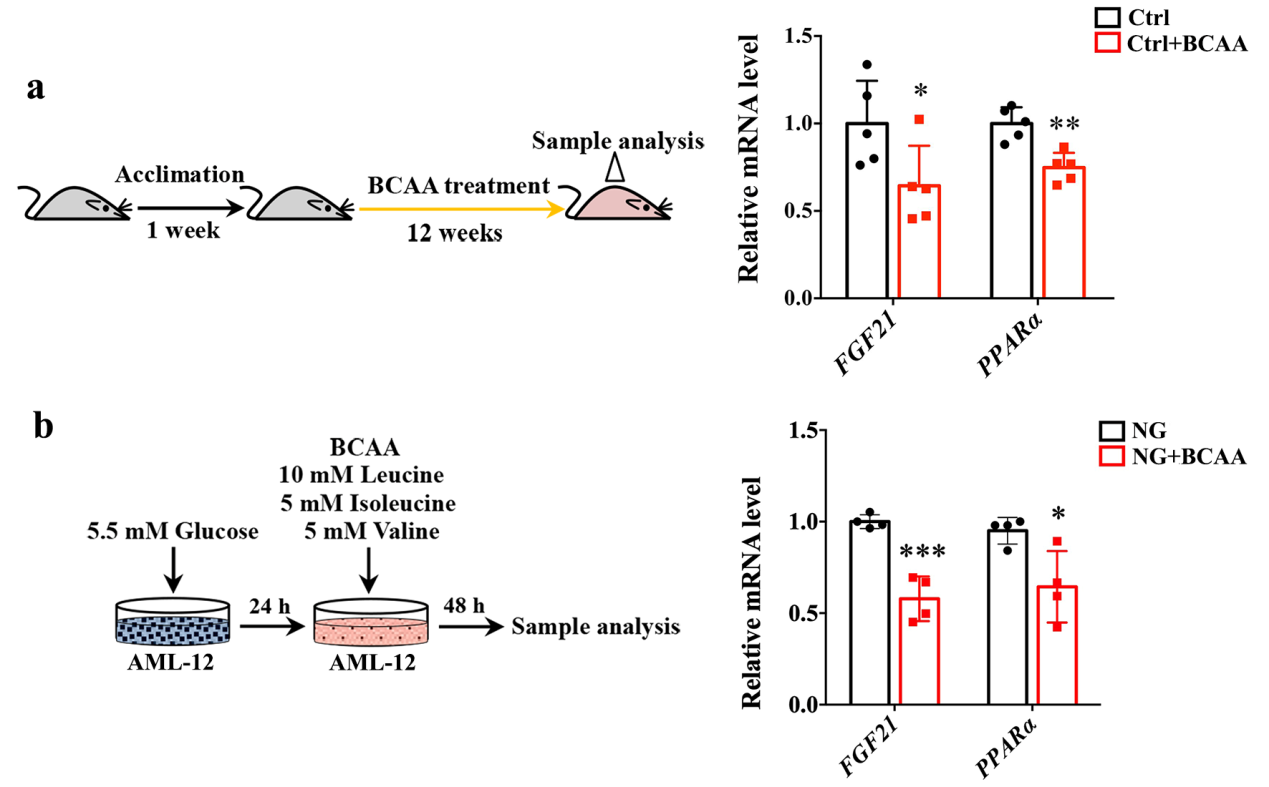


**Figure S10.** Excess BCAA decreases FGF21 production both in vivo and in vitro. (a) After 1 week of acclimation, mice were treated by intraperitoneal injection of BCAA for 12 weeks and then sacrificed for sample analysis. The relative mRNA levels of FGF21 and PPARα in the liver of control (Ctrl) mice after BCAA treatment. (b) Hepatocyte cell line AML-12 were cultured under a high BCAA condition (10 mM leucine, 5 mM isoleucine and 5 mM valine) for 48 h and then collected for sample analysis. The relative mRNA levels of FGF21 and PPARα in the hepatocyte under high BCAA condition. The difference between two groups was analyzed by using two-tailed unpaired student’s t test with Bonferroni correction. Significant level: *p<0.05; **p<0.01; ***p<0.001.


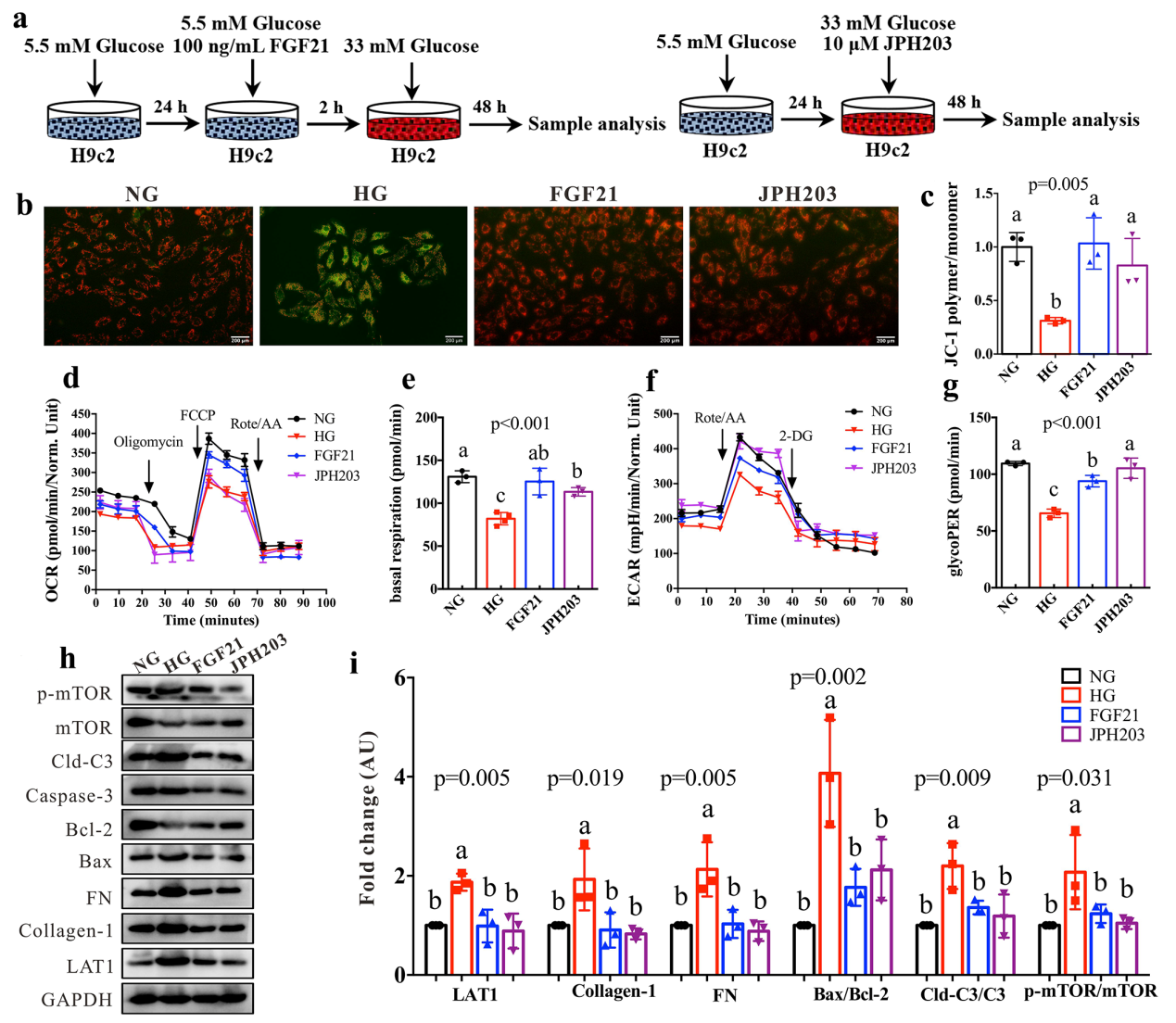


**Figure S11.** FGF21 alleviates mitochondrial damage and apoptosis of myocardial cells. (a) H9c2 cells were pretreated with FGF21 (100 ng/mL) for 2 h or treated with JPH203 (10 μM), cultured under a high glucose condition (33 mM glucose) for 48 h and then collected for sample analysis. (b) Representative images ofJC-1 staining and (c) the ratio of JC-1 polymer/monomer to indicate the mitochondrial membrane potential in H9c2 cells under normal glucose (NG) and high glucose (HG) conditions as well as FGF21- and JPH203-treated H9c2 cells under HG condition. (d) The curve of oxygen consumption rate (OCR) and (e) the corresponding quantitative data to indicate mitochondrial respiration in H9c2 cells under NG and HG conditions as well as FGF21- and JPH203-treated H9c2 cells under HG condition. (f) The curve of extracellular acidification rate (ECAR) and (g) the corresponding quantitative data to indicate mitochondrial glycolysis in H9c2 cells under NG and HG conditions as well as FGF21- and JPH203-treated H9c2 cells under HG condition. (h) Western blotting showing the expression levels of LAT1, Collagen-1, fibronectin (FN), Bax, Bcl-2, caspase-3, cleaved caspase-3 (Cld-C3), mTOR and p-mTOR in H9c2 cells under NG and HG conditions as well as FGF21- and JPH203-treated H9c2 cells under HG condition and (i) the corresponding quantitative results. The differences among four groups were analyzed by using one-way ANOVA with Bonferroni’s multiple comparisons test, and different lowercase codes represent a statistically significant difference (p<0.05).


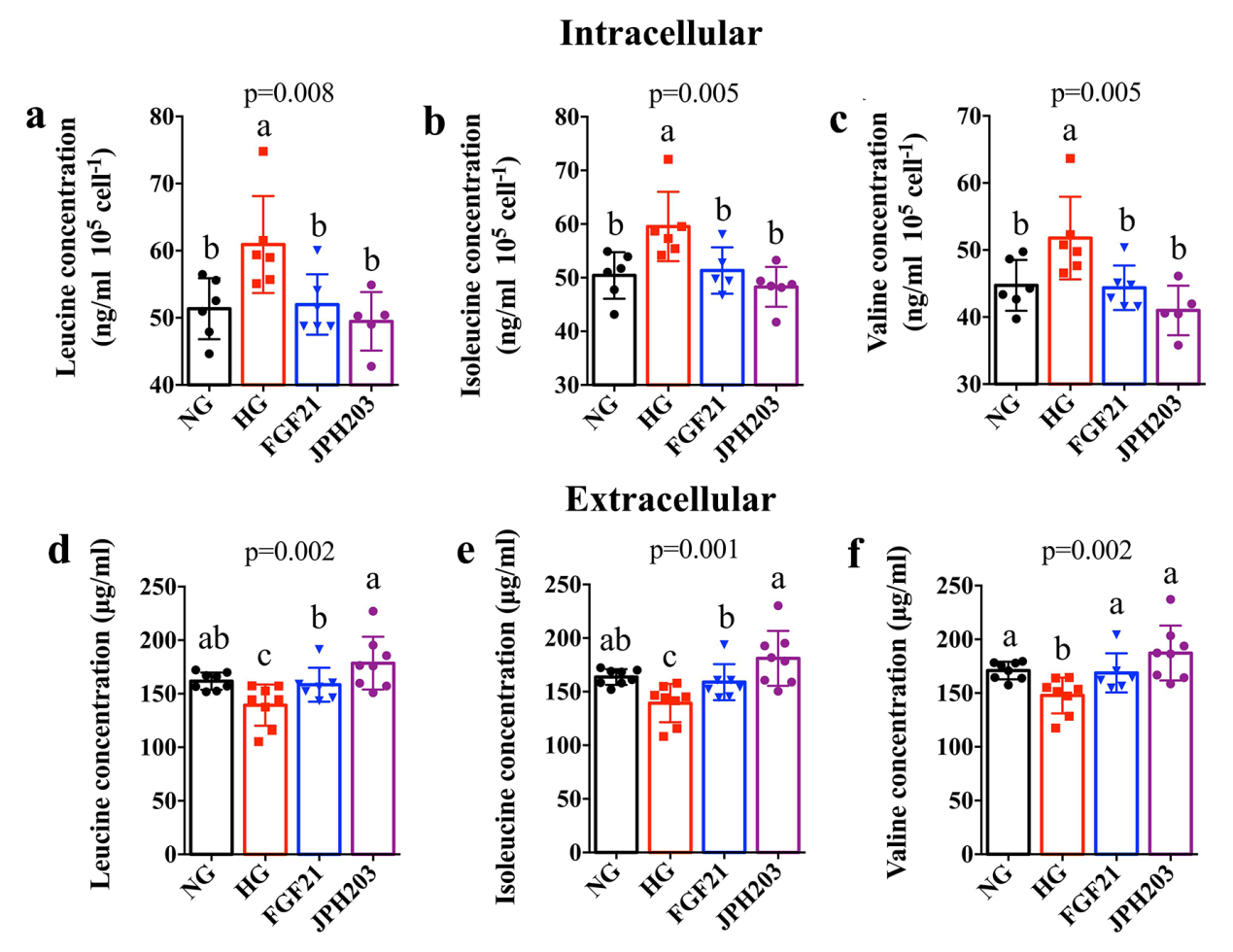


**Figure S12.** FGF21 reduces BCAA in H9c2 cells under high glucose condition. The (a-c) intracellular and (d-f) extracellular levels of leucine, isoleucine and valine in H9c2 cells under normal glucose (NG) and high glucose (HG) conditions as well as FGF21- and JPH203-treated H9c2 cells under HG condition. The differences among four groups were analyzed by using one-way ANOVA with Bonferroni’s multiple comparisons test, and different lowercase codes represent a statistically significant difference (p<0.05).


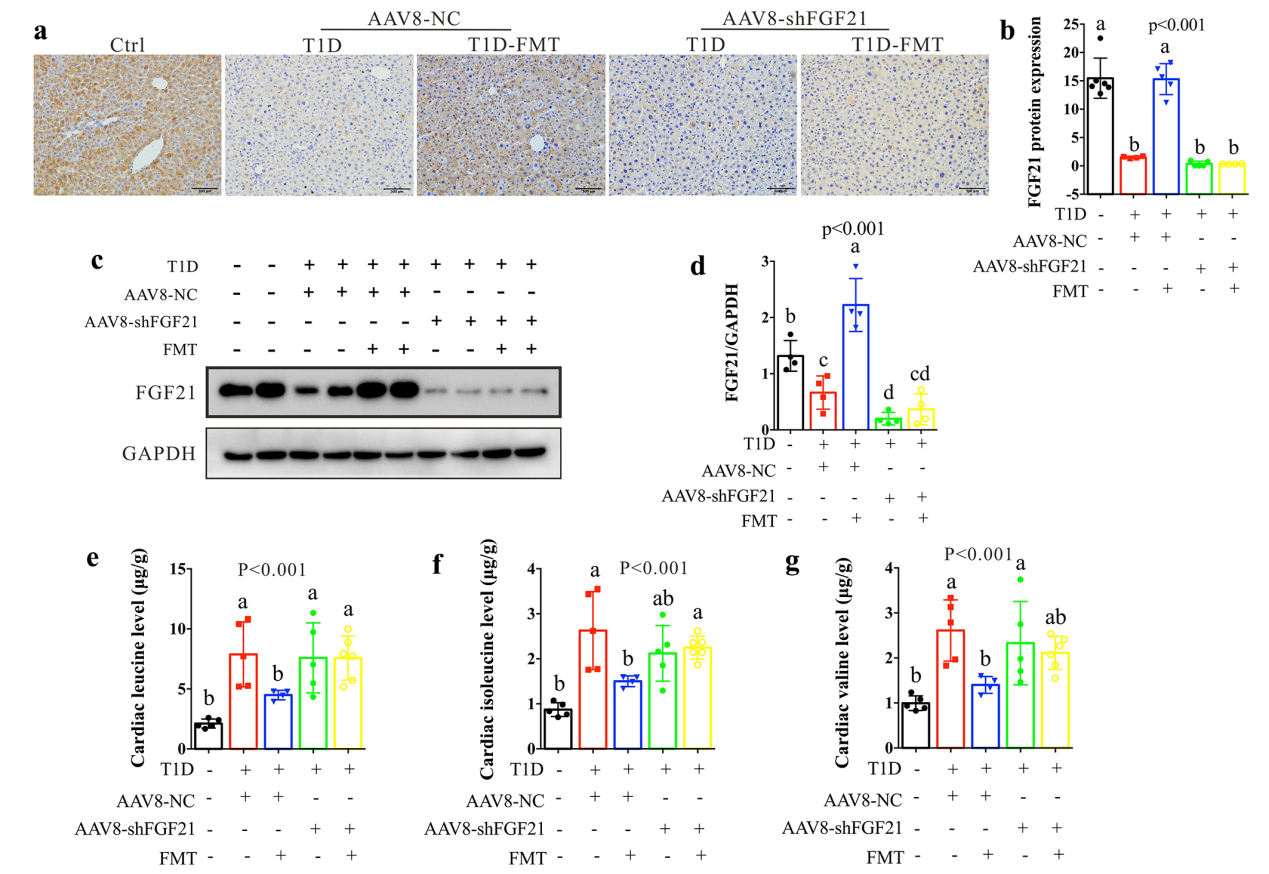


**Figure S13.** The effect of AAV-mediated FGF21 knockdown on hepatic FGF21 production and cardiac BCAA levels in T1D mice with FMT. (a) Representative histological images of FGF21 staining (bar=100 μm) and (b) the corresponding quantitative data to show the level of FGF21 in the liver of control (Ctrl) and T1D mice treated with AAV8-NC (empty vector) or AAV8-shFGF21 with and without FMT. (c) Western blotting showing the expression levels ofFGF21 in the liver of Ctrl and T1D mice treated with AAV8-NC or AAV8-shFGF21 with and without FMT and (d) the corresponding quantitative data. The levels of (e) leucine, (f) isoleucine and (g) valine in the heart of Ctrl and T1D mice treated with AAV8-NC or AAV8-shFGF21 with and without FMT. The differences among five groups were analyzed by using one-way ANOVA with Bonferroni’s multiple comparisons test, and different lowercase codes represent a statistically significant difference (p<0.05).
